# Age‐related changes in metabolites in young donor livers and old recipient sera after liver transplantation from young to old rats

**DOI:** 10.1111/acel.13425

**Published:** 2021-06-22

**Authors:** Qunhua Han, Hui Li, Mengyuan Jia, Lin Wang, Yulan Zhao, Mangli Zhang, Qin Zhang, Zhuoxian Meng, Jimin Shao, Yunmei Yang, Lijun Zhu

**Affiliations:** ^1^ Department of Geriatrics The First Affiliated Hospital Zhejiang University School of Medicine Hangzhou China; ^2^ Zhejiang Provincial Key Laboratory for Diagnosis and Treatment of Aging and Physic‐chemical Injury Diseases The First Affiliated Hospital Zhejiang University School of Medicine Hangzhou China; ^3^ Department of Pathology & Pathophysiology, and Cancer Institute of the Second Affiliated Hospital Zhejiang University School of Medicine Hangzhou China; ^4^ NHFPC Key Laboratory of Combined Multi‐Organ Transplantation The First Affiliated Hospital Zhejiang University School of Medicine Hangzhou China; ^5^ Zhejiang Provincial Key Laboratory of Pancreatic Disease The First Affiliated Hospital Zhejiang University School of Medicine Hangzhou China; ^6^ Department of Hepatobiliary and Pancreatic Surgery The First Affiliated Hospital Zhejiang University School of Medicine Hangzhou China; ^7^ Department of Pathology and Pathophysiology, and Key Laboratory of Disease Proteomics of Zhejiang Province Zhejiang University School of Medicine Hangzhou China; ^8^ Zhejiang University Cancer Center Hangzhou China

**Keywords:** liver ageing, metabolic pathways, orthotopic liver transplantation, transcriptomics, untargeted metabolomics

## Abstract

Liver ageing not only damages liver function but also harms systemic metabolism. To better understand the mechanisms underlying liver ageing, we transplanted the livers of young rats to young and old rats and performed untargeted metabolomics to detect changes in the metabolites in the liver tissues and sera. A total of 153 metabolites in the livers and 83 metabolites in the sera were different between the old and young rats that did not undergo liver transplantation; among these metabolites, 7 different metabolites were observed in both the livers and sera. Five weeks after liver transplantation, the levels of 25 metabolites in the young donor livers were similar to those in the old rats, and this result probably occurred due to the effect of the whole‐body environment of the older recipients on the young livers. The 25 altered metabolites included organic acids and derivatives, lipids and lipid‐like molecules, etc. In the sera, the differences in 78 metabolites, which were significant between the young and old rats, were insignificant in the old recipient rats and made the metabolic profile of the old recipients more similar to that of the young recipients. Finally, combining the above metabolomic data with the transcriptomic data from the GEO, we found that the altered metabolites and genes in the liver were enriched in 9 metabolic pathways, including glycerophospholipid, arachidonic acid, histidine and linoleate. Thus, this study revealed important age‐related metabolites and potential pathways as well as the interaction between the liver and the whole‐body environment.

## Graphical abstract

Aging is related to metabolic disorder. To better understand the underlying mechanisms, we transplanted livers of young rats to young and old rats. Analyses of differential metabolites in the livers and sera revealed that while the young donor livers underwent aging in the old recipients, they could benefit the old recipients through circulation. Integrated metabolomic and transcriptomic profiles suggested altered metabolic pathways during liver aging.

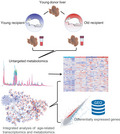

## 1 INTRODUCTION

The liver is a vital organ that performs metabolic functions that are essential for life, such as nutrient and energy metabolism, and its functions and status are critical to human health. Ageing is a major risk factor for liver disease and is related to the severity and poor prognosis of liver diseases (Kim et al., 2015; Ramirez et al., 2017). Liver disease not only damages liver function but also harms systemic metabolism. For example, nonalcoholic fatty liver disease (NAFLD) might progress to liver fibrosis, cirrhosis and eventually hepatocellular carcinoma; NAFLD is also a risk factor for type 2 diabetes mellitus, cardiovascular and cerebrovascular diseases, and chronic kidney disease (Anstee et al., 2013; Byrne & Targher, 2015). The enhancement of liver function can promote nerve regeneration in elderly mice and slow cognitive impairment through blood circulation (Horowitz et al., 2020). Therefore, it is of great significance to maintain the youth and health of the liver.

The liver has always been regarded as an organ that prevents ageing because of its regenerative ability. Liver function shows no obvious age‐related changes under nonpathological conditions. No discernible changes in gene expression were found in liver tissue when assayed by cDNA arrays (Zahn et al., 2007). Livers from octogenarian donors are acceptable for liver transplantation after careful selection (Cascales‐Campos et al., 2018; Gajate Martín et al., 2018), and liver cancer in patients over 90 years old can also be effectively treated by hepatectomy (Uwatoko et al., 2015). However, ageing is an inevitable process that is accompanied by a gradual reduction in liver volume and blood flow (Schmucker, 2005; Wynne et al., 1989). In addition, the ability of the liver to metabolize materials decreases, and the permeability of endothelial cells and the transfer and transportation of metabolites are impaired (Hilmer et al., 2005; Sheedfar et al., 2013), which in turn increase the risk of age‐related diseases of the liver itself and other organs of the body, and these diseases include NAFLD, diabetes, cardiovascular and cerebrovascular diseases, and tumours (Schmucker, 2005). Before the onset of the diseases, gene expression and metabolites may undergo broad changes. Using RNA‐seq and single‐cell sequencing, variations in cell type and mRNA expression were found in mouse livers with ageing (Consortium, T. T. M, 2020; Schaum et al., 2020; White et al., 2015). Energy dysregulation and ROS accumulation were detected by metabolomic study of aged rat liver (Son et al., 2012).

How to effectively delay or prevent age and age‐related diseases or even rejuvenate aged tissues and organs has always been an interesting research topic for scientists. When young and old mice share a circulatory system, the regeneration ability of aged liver progenitor cells is restored (Conboy et al., 2005), suggesting that young blood can improve aged liver function. However, an adult grafted liver shows no rejuvenation in a paediatric recipient based on senescence marker protein‐30 (SMP‐30) (Eguchi et al., 2011). The importance of changes in the liver itself and the internal environment of the body during the liver ageing process and how the liver and the body internal environment affect each other are not yet fully understood.

In this study, the livers of young donor rats were transplanted into young and old recipient rats. Five weeks after transplantation, liver and blood samples were collected, and untargeted metabolomics was performed. Then, we compared the metabolite changes in these samples from three perspectives: (1) differences in the liver tissues and sera between old and young rats not subjected to transplantation; (2) changes in the liver tissues of young donor rats 5 weeks after transplantation into old and young recipient rats; and (3) changes in the sera of old and young recipient rats after receiving young donor livers. Finally, by combined analysis with the RNA‐seq transcriptomics data of young and old rats from the Gene Expression Omnibus (GEO) database, a network of liver age‐related metabolites and genes was explored.

## 2 RESULTS

### 2.1 Rats were in good general condition 5 weeks after liver transplantation

**FIGURE 1:**
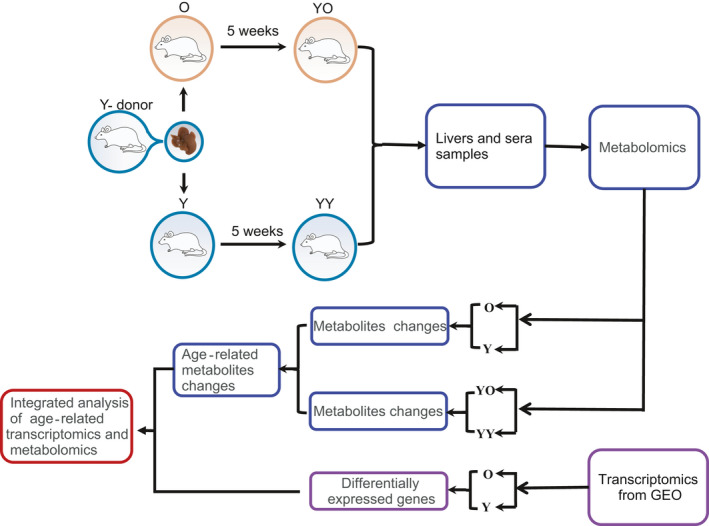
Overall experimental design and analysis workflow. Livers of young donors (Y, 3–5 months) were transplanted into young (Y, 3–5 months, *n* = 8) and old (O, 15–17 months, *n* = 8) recipients. After OLT, the young and old recipients were named the YY and YO groups. Liver and blood samples collected during OLT from young and old rats were used as O and Y samples. The liver and blood samples collected 5 weeks after OLT were used as YO and YY samples. Blood sample was drawn from the inferior vena cava

**FIGURE 2:**
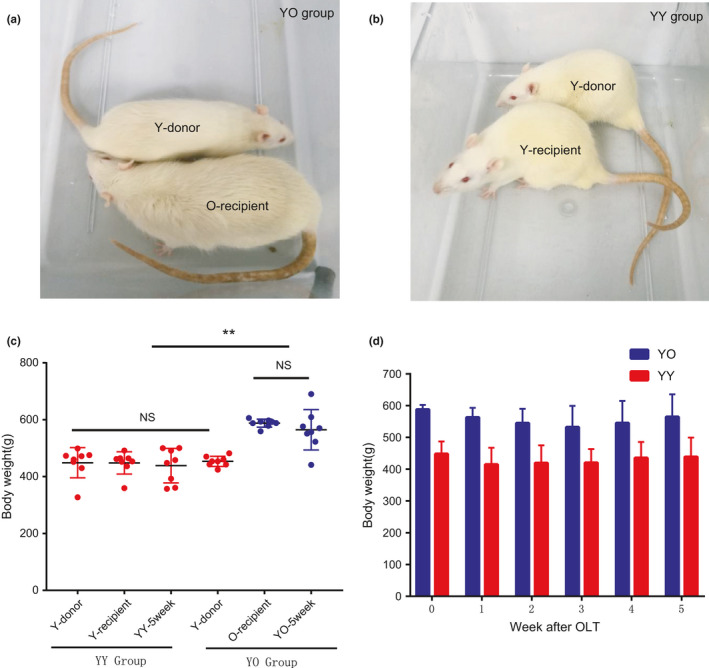
Rat appearance and bodyweight. (a, b) Representative photographs of donor and recipient rats before OLT. Young rats were lively with smooth and shiny hair, while the old rats were larger in body size and had less hair. (c) Body weights of different groups of rats. The Y‐donor, Y‐recipient and O‐recipient groups were weighed before OLT, and the YO and YY rats were weighed before sampling at 5 weeks after OLT. (d) Weekly bodyweight of YY and YO rats after OLT. The body weight of the YO group decreased modestly after OLT and then gradually recovered. No obvious change in body weight was observed in the YY group after OLT. No significant differences were observed in the body weight between the YO or YY groups. The data were shown as the mean ± SD. Significance was analysed by one‐way ANOVA. NS represented no significance, **represented *p* < 0.01

Using the two‐cuff orthotopic liver transplantation (OLT) technique, the livers of young rats (3–5 months) were transplanted into old rats (15–17 months) and young rats (3–5 months) (Figure 1). Representative photographs of donor and recipient rats before transplantation are shown in Figure 2. The young rats (Y) were lively with smooth and shiny hair, while the old rats (O) were larger in body size and had less hair. In the first few days after OLT surgery, the recipient rats showed reduced energy, less activity, reduced food intake and lower body weight. The activity of the recipient rats was obviously restored 3 days after successful OLT. The body weights of the old recipient rats (YO) decreased moderately after OLT but gradually recovered to presurgery levels at the 5th week. All the rats used for subsequent experiments were in good general condition at the 5th week after liver transplantation (Movies S1 and S2). No obvious change in body weight was observed in young recipient rats (YY) after OLT (Figure 2).

Haematoxylin‐eosin (HE) staining of rat liver tissues showed clear hepatic lobules, radial liver cell cords and clear sinusoids in the Y group. Although the sinusoids of the O group were not as clear as that of the Y group, the overall structures of liver tissue were well preserved. HE staining also revealed normal hepatic architectures with central veins and radiating hepatic cords similarly in the YY and YO groups (Figure S1a). Concentrations of glucose (Glu), total cholesterol (TC), triglycerides (TG), high‐density lipoprotein (HDLC) and low‐density lipoprotein (LDLC) were also measured in the rat sera (Figure S1b). No significant differences were observed between O and Y groups and between YO and YY groups.

### 2.2 Metabolic profiling of the liver tissues and sera of rats before and after liver transplantation

Untargeted metabolomics analysis was performed using LC/MS after sample extraction. Both negative and positive modes of mass spectra were examined. For metabolites in the liver tissues, 12,993 distinct features in positive mode and 13,596 features in negative mode were detected. In total, approximately 10.3% of features in positive mode and 11.4% of features in negative mode were different between O and Y liver tissues (Figure S2a,b), while approximately 2.7% of features in positive mode and 3.0% of features in negative mode were different between YO and YY liver tissues (Figure S2c,d). After combining positive and negative mode features, principal component analysis (PCA) of metabolites showed a separated distribution between O and Y liver tissues. Notably, a small change was observed in the metabolites of YO and YY liver tissues, suggesting a different influence of the body environments of the old and young recipient rats on the young donor livers (Figure S4a,b).

For serum metabolites, 12,102 features were detected in the positive mode, and 6,681 features were detected in the negative mode. Approximately 11% of features in positive mode and 11% of features in negative mode between O and Y sera (Figure S3a,b) and approximately 6.5% of features in positive mode and 4.7% of features in negative mode between YO and YY sera were different (Figure S3c,d). PCA showed more similar pattern of metabolites in the sera between the YO and YY compared with the significant difference between Y and O (Figure S4c,d), suggesting that the changed serum metabolites of the old rats were reduced after receiving a young liver.

### 2.3 The levels of twenty‐five metabolites in young livers became similar to those of old rats after transplantation into old recipient rats

**FIGURE 3:**
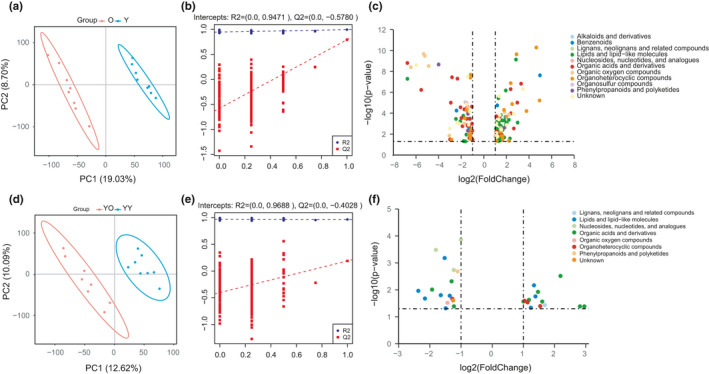
Metabolite changes in liver tissues. (a, b) PLS‐DA plot of the O and Y groups. (d, e) PLS‐DA plot of the YO and YY groups. (b, e) Validation of the PLS‐DA model by the 200‐time permutation test. (c) Volcano plot of different metabolites between the O and Y groups. (f) Volcano plot of different metabolites between the YO and YY groups

A supervised multivariate statistical analysis was performed using partial least squares‐discriminant analysis (PLS‐DA) to screen differential metabolites. A significant separation in liver tissues between the O and Y group, YO and YY group was observed in the PLS‐DA plots (Figure 3a,d). From the 200 permutation tests, the R2 and Q2 values of different groups demonstrated that the PLS‐DA model was reliable without overfitting (Figure 3b,e).

We analysed annotated metabolites in liver tissues and found 153 differential metabolites between the O and Y group (VIP >1, *p* < 0.05, ratio >2 or ratio <0.5), among which 70 metabolites were downregulated (O/Y ratio <0.5) and 83 metabolites were upregulated (O/Y ratio >2) (Table S1‐1). The top altered metabolites were lipids and lipid‐like molecules (45 metabolites), organic acids and derivatives (34 metabolites), organoheterocyclic compounds (26 metabolites), and organic oxygen compounds (13 metabolites) (Figure 3c, Table S1‐1). The majority of fatty acyl and glycerophospholipid metabolites, especially lysoglycerophospholipids, increased. Most of the decreased metabolites were carboxylic acids, keto acids, and their derivatives. The enrichment analysis of KEGG pathways showed that the differential metabolites were enriched in metabolic pathways, especially in glycerophospholipid metabolism (Figure S5a).

At 5 weeks after transplantation, 31 differential metabolites in the young donor livers were identified between the YO and YY groups (Figure 3f, Table S1‐2). When comparing the altered metabolites in liver tissues between the O/Y (153 metabolites) and YO/YY groups, 15 metabolites were found to be different in both the YO/YY and O/Y groups, 10 metabolites that met the change criteria (VIP >1, *p* < 0.05, ratio >2 or ratio <0.5) in the O/Y group met only two of the three criteria (*p* < 0.05, VIP >1), but the ratio was between 0.5–2 in YO/YY, and 128 metabolites that were significantly different in the O/Y group were insignificantly different in the YO/YY group (*p* > 0.05). The 25 metabolites showed the same alterative trend (both increased or decreased) in the O/Y and YO/YY groups, and the levels of the metabolites in the young donor livers of the YO group became similar to the levels of the metabolites in the livers of old rats (Table 1, Figure S6). The observations suggested an interaction between the young transplanted liver and the old recipient body; probably, the internal environment of the old body made the young liver tend to age. In other words, these 25 metabolites might correlate with liver ageing.

**TABLE 1:** Twenty‐five differential metabolites in O/Y and YO/YY of liver tissues

Metabolite classification		Metabolite	O/Y			YO/YY			KEGG pathway
Superclass	Class		ratio	*p* value	VIP	ratio	*p* value	VIP	
Organic acids and derivatives	Carboxylic acids and derivatives	Cyclo(proline‐leucine)	7.71	0.0000	3.40	2.00	0.0270	2.56	
		Ergothioneine	2.12	0.0060	1.67	1.62	0.0335	1.53	Histidine metabolism Metabolic pathways
		Hydroxyprolyl‐Lysine	6.22	0.0029	2.39	2.77	0.0119	2.53	
	Keto acids and derivatives	(S)‐2‐Aceto‐2‐hydroxybutanoic acid	5.23	0.0001	2.90	1.79	0.0013	1.43	Valine, leucine and isoleucine biosynthesis Metabolic pathways 2‐Oxocarboxylic acid metabolism Biosynthesis of amino acids
		2‐Keto−6‐aminocaproate	9.71	0.0000	3.53	3.04	0.0272	2.45	Lysine degradation Metabolic pathways
		Ethyl 3‐oxohexanoate	0.01	0.0000	4.84	0.26	0.0098	1.83	
	Organic sulphuric acids and derivatives	Phenol sulphate	0.02	0.0000	4.00	0.43	0.0410	1.81	
Lipids and lipid‐like molecules	Fatty Acyls	Isopropyl tiglate	0.18	0.0004	2.73	0.35	0.0007	2.92	
	Glycerophospholipids	PG 6:0; PG(2:0/4:0)	0.01	0.0000	4.70	0.32	0.0158	1.55	Glycerophospholipid metabolism Metabolic pathways
	Prenol lipids	4‐Hydroxy−3‐polyprenylbenzoate	7.10	0.0000	3.24	2.53	0.0068	2.37	
		3,7‐Dihydroxy−12‐oxocholanoic acid	0.29	0.0467	1.78	0.19	0.0109	3.03	
Organic oxygen compounds	Organooxygen compounds	1‐Hydroxy−2‐pentanone	3.52	0.0001	2.44	1.62	0.0013	1.35	
		3‐Keto‐b‐D‐galactose	0.01	0.0000	4.81	0.37	0.0303	1.46	
		xi−5‐Acetyltetrahydro−2(3H)‐furanone	4.64	0.0001	2.75	1.76	0.0010	1.40	
Organoheterocyclic compounds	Diazines	2,6‐Dimethylpyrazine	5.62	0.0000	2.79	1.68	0.0423	2.17	
	Dihydrofurans	xi−2,3‐Dihydro−3‐methylfuran	3.91	0.0002	2.46	1.85	0.0287	1.83	
	Pyridines and derivatives	Pyridoxamine	3.41	0.0000	2.44	2.19	0.0292	2.08	Vitamin B6 metabolism Metabolic pathways Vitamin digestion and absorption
Nucleosides, nucleotides, and analogues	Purine nucleosides	Deoxyinosine	0.41	0.0050	1.80	0.43	0.0018	2.34	Purine metabolism Metabolic pathways ABC transporters
		Inosine	0.32	0.0000	1.89	0.53	0.0036	1.71	Purine metabolism Metabolic pathways ABC transporters
Lignans, neolignans and related compounds	Furanoid lignans	Enterolactone	2.10	0.0270	1.66	3.22	0.0369	2.62	
Organosulphur compounds	Organic disulphides	Methyl 3‐methyl‐1‐butenyl disulphide	0.32	0.0000	2.52	0.59	0.0009	1.51	
Phenylpropanoids and polyketides	Flavonoids	Eriodictyol	0.37	0.0046	1.99	0.46	0.0021	2.05	Metabolic pathways
–	–	13,14‐Dihydro‐16,16‐difluoroprostaglandin D2	2.30	0.0169	1.44	1.99	0.0277	1.60	
		3‐Aminohexanoic acid	10.82	0.0000	3.80	1.99	0.0370	2.24	
		N‐Phthalyl‐L‐tryptophan	0.02	0.0000	4.71	0.42	0.0247	1.21	

### 2.4 The differences in ninety‐four percent of the altered serum metabolites in old rats became insignificant after young liver transplantation

**FIGURE 4:**
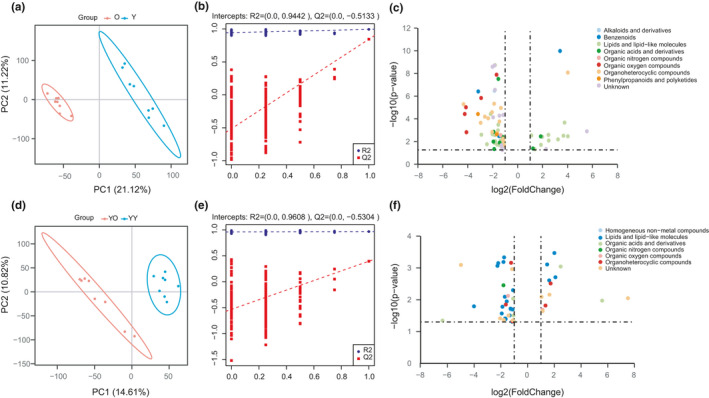
Changes in sera metabolites. (a, b) PLS‐DA plot of the O and Y groups. (d, e) PLS‐DA plot of the YO and YY groups. (b, e) Validation of the PLS‐DA model by the 200‐time permutation test. (c) Volcano plot of different metabolites between the O and Y groups. (f) Volcano plot of different metabolites between the YO and YY groups

A total of 83 different metabolites (VIP >1, *p* < 0.05, ratio >2 or ratio <0.5) were identified in the sera between the O and Y groups (Figure 4, Table S2‐1), among which approximately 80% were downregulated. The 83 different metabolites mainly belonged to the superclasses of lipids and lipid‐like molecules (40%), organoheterocyclic compounds (17%), and organic acids and derivatives (10%). Among the 33 changed metabolites in the superclass of lipids and lipid‐like molecules, 14, 9, and 7 were sterol lipids, fatty acyls, and glycerophospholipids, respectively. Interestingly, 13 different metabolites among sterol lipids were bile acids. All these different metabolites were enriched in the KEGG pathways of glycerophospholipid metabolism, linoleic acid metabolism and phenylalanine metabolism (Figure S5b).

There were 44 different metabolites in the sera between the YO and YY groups. Similar to the difference between the O and Y groups, the altered metabolites were also mainly downregulated (70%), and 45% of the altered metabolites were lipids and lipid‐like molecules (Figure 4f, Table S2‐2).

**FIGURE 5:**
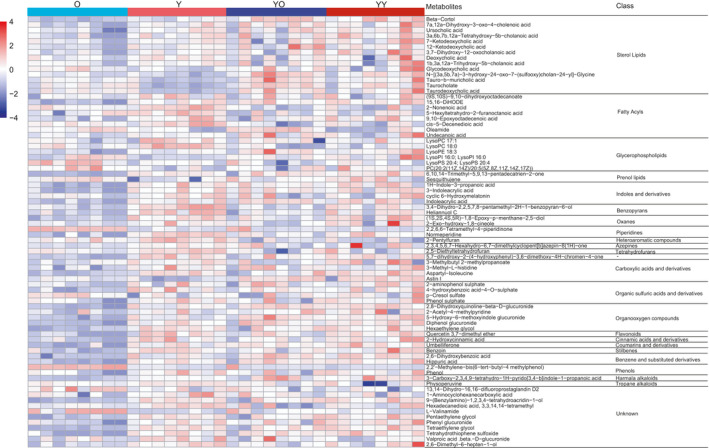
Heatmap of 78 metabolites that significantly different in the sera of the O and Y groups but were insignificantly different in the sera of the YO and YY groups. The colour scale bar (upper left of heatmap) shows the range of levels of metabolites: red indicates high, blue indicates low

When comparing changed metabolites in sera between the O/Y (83 metabolites) and YO/YY (44 metabolites) groups, only 5 of the metabolites, including (3beta,8beta)‐3‐hydroxy‐7(11)‐eremophilen‐12.8‐olide, 1‐(1Z‐octadecenyl)‐sn‐glycero‐3‐phosphocholine, docosahexaenoic acid methyl ester, hecogenin, and trimethylamine N‐oxide, were found to be shared by the two paired groups of rats. Most surprisingly, among the 83 altered metabolites in the sera between the O and Y group, 78 (94%) of the metabolites did not change significantly between the YO and YY groups (75 metabolites *p* > 0.05 and 3 metabolites *p* < 0.05 but ratio between 0.5–2). None of the 13 bile acid metabolites that were significantly altered between the O and Y groups showed a significant change between the YO and YY groups. Figure 5 showed that the profiles of the 78 metabolites were similar to the profiles of the 78 metabolites of young rats. The results suggest that the transplanted young livers had a great influence on the metabolism of the old recipient rats.

### 2.5 Differences in five metabolites in both the livers and sera of old rats became insignificant after the old rats received young livers

The liver is rich in blood supply. Nutrients and harmful substances can be transported into the liver for further processing and then enter systemic circulation. By comparing the differential metabolites in livers (153 metabolites) and sera (83 metabolites) between the O and Y groups, 7 differential metabolites were identified both in the livers and sera of rats; 3 metabolites (taurodeoxycholic acid, lysoPS 20:4, and13,14‐dihydro‐16,16‐difluoroprostaglandin D2) were increased, and 4 metabolites (3,7‐dihydroxy‐12‐oxocholanoic acid, hippuric acid, phenol sulphate, and phenyl glucuronide) were decreased (Table 2). Interestingly, 5 of the 7 differential metabolites were not significantly altered in the liver tissues and sera between the YO and YY groups. The results further illustrated the interaction between the livers and whole‐body environments through circulation.

**TABLE 2:** Differential metabolites of O/Y in serum and liver

Metabolite classification		Metabolite name	serum			liver		
Superclass	Class		ratio	*p* value	VIP	ratio	*p* value	VIP
Lipids and lipid‐like molecules	Sterol Lipids	3,7‐Dihydroxy‐12‐oxocholanoic acid	0.40	0.0198	1.48	0.29	0.0467	1.78
		Taurodeoxycholic acid	16.25	0.0031	2.40	2.57	0.0270	1.43
	Glycerophospholipids	LysoPS 20:4	5.53	0.0023	1.96	7.85	0.0008	2.64
Benzenoids	Benzene and substituted derivatives	Hippuric acid	0.11	0.0000	3.20	0.40	0.0025	1.58
Organic acids and derivatives	Organic sulphuric acids and derivatives	Phenol sulphate	0.37	0.0114	1.77	0.02	0.0000	4.00
‐	‐	Phenyl glucuronide	0.40	0.0195	1.64	0.12	0.0028	2.36
		13,14‐Dihydro‐16,16‐difluoroprostaglandin D2	4.85	0.0418	2.02	2.30	0.0169	1.44

### 2.6 Network analyses for the differential metabolites and genes involved in liver ageing

To further study the mechanisms underlying the metabolite changes related to ageing, RNA‐seq transcriptomics data from the liver tissues of 4 young (21‐week‐old) male rats and 4 old (104‐week‐old) male rats were downloaded from the Gene Expression Omnibus (GEO) database (accession number GSE53960) (Yu et al., 2014). A total of 780 differentially expressed genes (398 increased and 382 decreased) were identified between the liver tissues of the old and young rats (Figure S7a, Table S3). These altered genes were abundantly enriched in metabolic pathways and overlapped with the pathways associated with the altered metabolites of the O and Y groups, such as glycerophospholipid metabolism and linoleic acid metabolism (Figures S5a and S7b).

**FIGURE 6:**
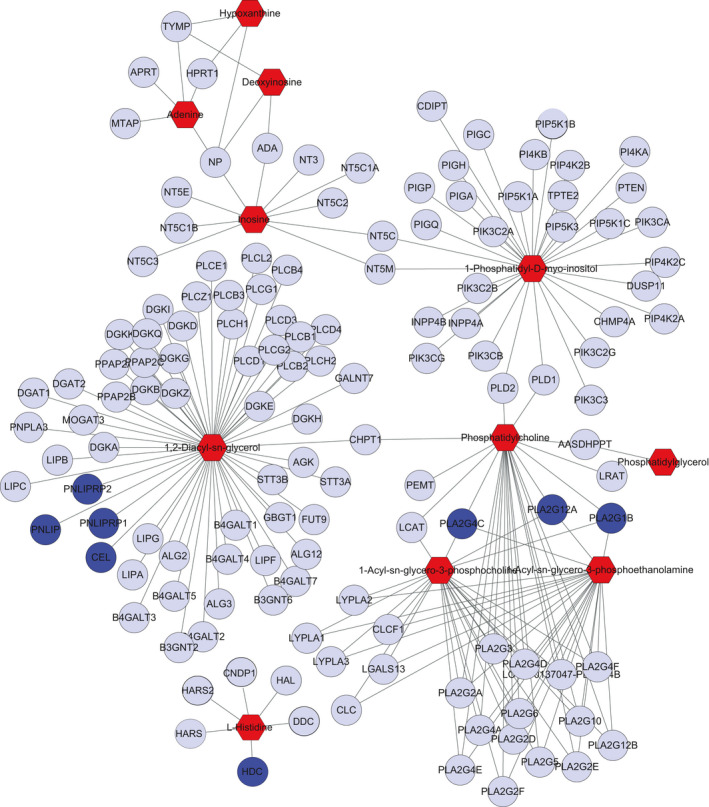
Part of the closely connected network of metabolites and genes. The nodes in red indicate differential metabolites, and the nodes in blue indicate differentially expressed genes. Hexagons represent metabolites, circles represent genes. The fully connected network of metabolites and genes is shown in Figure S8

We further used Metscape to analyse context relationships between the altered metabolites and genes in the livers, which formed an intricate network (Figure 6, Figure S8). Among these metabolites and genes, 9 metabolism pathways (glycerophospholipid metabolism, arachidonic acid metabolism, histidine metabolism, linoleate metabolism, bile acid biosynthesis, butanoate metabolism, lysine metabolism, phosphatidylinositol phosphate metabolism, and purine metabolism) included both changed genes and metabolites. Differentially expressed genes in 4 pathways (glycerophospholipid metabolism, arachidonic acid metabolism, histidine metabolism, linoleate metabolism) were directly related to changed metabolites.

Figure 6 showed some of the altered metabolites and closely related differentially expressed genes. Glycerophospholipids (phosphatidylcholine) and lysophospholipids (1‐acyl‐sn‐glycero‐3‐phosphocholine, 1‐acyl‐sn‐glycero‐3‐phosphoethanolamine) were closely linked with phospholipaseA2 (Pla2g1b, Pla2g12a, and Pla2g4c). Indeed, Pla2 preferentially hydrolyses the ester bond of the fatty acyl group attached to the sn‐2 position of phospholipids, producing lysophospholipids and fatty acids. Ageing possibly leads to the accumulation of glycerophospholipids and the alteration of Pla2 expression, which in turn leads to an increase in lysophospholipids. Figure 6 also showed that changed genes (histidine decarboxylase, HDC) and changed metabolites (L‐histidine) were associated with each other. HDC catalyses histidine and transforms into histamine in the histidine metabolism pathway. These results may explain the metabolic mechanisms related to liver ageing.

## 3 DISCUSSION

Ageing is the major risk factor for many age‐related diseases such as NAFLD, type 2 diabetes mellitus, cardiovascular and neurodegeneration diseases, and cancer. Nine hallmarks that contribute to the ageing process have been proposed: genomic instability, telomere attrition, epigenetic alterations, loss of proteostasis, deregulated nutrient sensing, mitochondrial dysfunction, cellular senescence, stem cell exhaustion, and altered intercellular communication (Lopez‐Otin et al., 2013). Each of the nine hallmarks of ageing is related to metabolic disorders (López‐Otín et al., 2016). The liver plays a central role in metabolism. Many metabolic processes, such as synthesis, decomposition and transformation, are carried out in the liver. Metabolic disorders of the liver can cause multiple organ and multisystem insufficiencies. As final products of various biological processes, metabolites have been regarded as the ultimate indicator of genetic or environmental changes in biological systems. Analyses of the metabolite changes related with liver ageing may help to understand the mechanisms of metabolic disorders and age‐related diseases, thereby providing novel biomarkers or targets for preventing or delaying ageing.

Orthotopic liver transplantation is a powerful research method for investigating liver biology, tissue damage, immunity and specific liver disease pathogenesis (Yokota et al., 2016). OLT facilitates the study of interactions between the liver and the internal environment of the body. We transplanted young livers into old rats and detected metabolite changes in the transplanted livers and the sera of recipient rats 5 weeks later. Metabolomics has emerged as a useful tool to identify novel diagnostic molecules and to develop new therapeutic targets for a variety of diseases because of its high sensitivity and capability to simultaneously detect multiple metabolites (Mato et al., 2019; Patel & Ahmed, 2015; Perakakis et al., 2020). In this study, to profile the metabolic changes and understand the underlying molecular mechanisms related to ageing, untargeted metabolomics was used to detect differential metabolites in the livers and sera of young and old rats as well as young and old recipient rats transplanted with young donor livers. We further downloaded the liver tissue transcriptomic data of young and old rats from the GEO database for age‐related differentially expressed genes. Finally, Metscape analyses integrated the metabolomics and transcriptomics data and showed that the both altered metabolites and related genes were associated with nine pathways, especially glycerophospholipid metabolism, arachidonic acid metabolism, histidine metabolism, and linoleate metabolism. Among these pathways, some altered genes were exactly the key enzymes that regulate the metabolites. For example, Pla2 can hydrolyse phospholipids producing lysophospholipids and fatty acids (Murakami et al., 2017), pnlip hydrolyses triacylglycerol and is involved in lipid metabolism (Gyetvai et al., 2017), and HDC can transform histidine into histamine (Hirasawa, 2019). These results suggested that the metabolic balance of glycerophospholipid, arachidonic acid, histidine, and linoleate loss was importantly involved in liver ageing.

Compared with younger liver tissues, 153 metabolites were significantly altered in old liver tissues, and 29% of these metabolites were lipids and lipid‐like molecules; the metabolites in this category, especially glycerophospholipids and lysophospholipids, were mainly increased. Interestingly, transcriptomic analysis revealed that the expression of Pla2g12a, Pla2g4c, and Pla2g1b was changed in aged liver tissue. Pla2 plays a key role in a variety of cellular responses, including phospholipid digestion and metabolism, host defence, and signal transduction. Pla2 catalyses glycerophospholipids to produce arachidonic acid (AA), a precursor of prostaglandins (PGs) and leukotrienes (LTs), as well as lysophospholipids. Overproduction of these metabolites causes inflammation and liver disease (Murakami et al., 2017). In addition, 22% of the altered metabolites were organic acids and derivatives, and these metabolites were decreased, suggesting that the digestion and absorption of nutrients by the ageing liver is reduced.

We found that 25 metabolites in the young donor liver became similar to those of old rat after transplantation into the old body (Table 1). The result suggested that the 25 metabolites were related to liver ageing due to the influence of the old recipient body environment on the young donor liver, probably accelerating the ageing of the young liver. The altered metabolites and related genes were mapped to KEGG pathways using Metscape. By analysing these pathways, we can identify the upstream and downstream relationship between the altered metabolites and altered genes and better understand the mechanism of liver ageing.

Serum metabolites were also altered in aged rats. Eighty‐three metabolites were significantly different between the O and Y groups, and 40% of the changed metabolites were lipids and lipid‐like molecules. Intriguingly, the most altered metabolites in this category were sterol lipids, and 13 among 14 sterol lipids were bile acids (BAs). BAs are the end products derived from cholesterol catabolism in the liver and released into the small intestine for the metabolism and absorption of lipids (Krøll, 2012). BAs perform a variety of biological functions and play an important regulatory role in metabolism and inflammation by interacting with several receptors (Krøll, 2012; Li et al., 2017). Studies have shown that calorie restriction, a well‐known anti‐ageing intervention, can alter serum bile acid concentrations (Fu & Klaassen, 2013). Moreover, bile acid synthesis and bile flow are markedly reduced during ageing (Krøll, 2012). Notably, after receiving young livers, 78 metabolites among the 83 altered serum metabolites in old rats became insignificant. This result suggested that while the young donor livers underwent ageing in the old recipients, they could change the metabolism of the old recipients, benefiting the whole body through circulation.

In conclusion, this study presented the metabolite profiles and pathways related to liver ageing and demonstrated the interaction between the liver and body environment with an OLT rat model and untargeted metabolomics combined with GEO transcriptomic data. We revealed that the metabolism by which the balances in glycerophospholipids, arachidonic acid, histidine, and linoleate are lost with liver ageing. Moreover, we found that 25 metabolites might accelerate liver ageing and might be potential biomarkers of liver ageing. Most of the serum metabolites, especially bile acids, were significantly altered with ageing, but these differences became insignificant after transplant of a young liver, indicating an important function of the liver in maintaining the health of the whole body. Further validation of the altered metabolites and related genes and analyses of the underlying detailed mechanisms in the future may provide novel biomarkers or targets for preventing or delaying ageing.

## 4 EXPERIMENTAL PROCEDURES

### 4.1 Animals

Male Sprague‐Dawley (SD) rats were purchased from Beijing Vital River Laboratory Animal Tech (Beijing, China). SD rats were housed under a 12h:12h light:dark cycle and were given ad libitum access to food and water in the Experimental Animal Center (First Affiliated Hospital, School of Medicine, Zhejiang University). Young (3–5 months) and old (15–17 months) rats were used in this study. All the experiments with animals were approved by an animal committee for ethics of the First Affiliated Hospital, College of Medicine, Zhejiang University. All the experiments were performed in accordance with relevant guidelines and regulations.

### 4.2 Experimental design

The overall experimental design is outlined in Figure 1. Young rats (3–5 months) were randomly divided into the Y‐donor (young donor) and Y‐recipient (young recipient) groups, and old rats (15–17 months) were included in the O‐recipient (old recipient) group. After liver transplantation, the Y recipient and O recipient groups were named the YY and YO groups, respectively. The YY and YO rats were observed daily, and dead or moribund rats were discarded or euthanized. Five weeks after liver transplantation, 8 young rats and 8 old rats, which were in good physical condition, were sacrificed. Blood and tissue samples were used for untargeted metabolomics analysis.

### 4.3 Rat orthotopic liver transplantation

Orthotopic liver transplantation (OLT) was performed using the cuff method as previously described (Jia et al., 2015). In brief, both donor and recipient rats were fasted for 12 h before surgery. The donor was anaesthetized by intraperitoneal injection of 4% chloral hydrate, and the liver was isolated and perfused through the portal vein with cold saline containing 25 U/ml heparin. The graft was preserved in 4°C University of Wisconsin (UW) solution before being transplanted into the recipient. The recipient liver was carefully removed after anaesthesia. The graft liver was orthotopically placed, and anastomosis of the suprahepatic vena cava was performed. After portal vein cuff connection, the liver restored blood flow. Then, bile duct reconstruction was carried out. Finally, the skin was sutured, and the rat recovered from anaesthesia.

### 4.4 Sample collection, storage and preparation

During OLT, 1 ml of blood was drawn from the inferior vena cava as a blood sample. After washing with saline solution, the liver tissue was cut into small pieces and quickly frozen in liquid nitrogen. The blood was centrifuged at 2,000 *g* for 20 min, and the supernatant was stored at −80°C.

Metabolites were extracted separately from the liver tissue or serum with 50% methanol buffer. A total of 100 mg liver tissue was ground in liquid nitrogen. Serum samples were thawed on ice. Twenty microlitres of serum or 100 mg of ground tissue was extracted with 120 μl of precooled 50% methanol, vortexed for 1 min, and incubated at room temperature for 10 min; the extraction mixture was then stored overnight at −20°C. After centrifugation at 4,000 *g* for 20 min, the supernatants were transferred into new 96‐well plates. The samples were stored at −80°C prior to LC/MS analysis. In addition, pooled quality control (QC) samples were also prepared by combining 10 μl of each extraction mixture.

### 4.5 Chromatography and mass spectrometry

All chromatographic separations were performed using an ultraperformance liquid chromatography (UPLC) system (SCIEX, UK). A TripleTOF5600plus high‐resolution tandem mass spectrometer (SCIEX, UK) was used to detect metabolites eluted from the column. The Q‐TOF was operated in both positive and negative ion modes. During the acquisition, the mass accuracy was calibrated every 20 samples. To verify and maintain data quality, samples were analysed in random order with QC samples analysed every 8 samples in the data acquisition sequence. LC/MS and untargeted metabolomics raw data were performed at LC‐Bio Technology Co., Ltd, Hangzhou, Zhejiang Province, China.

### 4.6 Untargeted metabolomics analyses

LC/MS raw data files were converted into mzXML format and then processed by the XCMS, CAMERA and metaX toolboxes implemented with R software. Each ion was identified by combining retention time and m/z data. The online KEGG and HMDB databases were used to annotate the metabolites by matching the exact molecular mass data (m/z) of samples with those from the database. An in‐house fragment spectrum library of metabolites was also used to validate metabolite identification. The intensity of the peak data was further preprocessed by metaX. PCA was performed for outlier detection and batch effect evaluation using the preprocessed dataset. Quality control‐based robust LOESS signal correction was fitted to the QC data with respect to the order of injection to minimize signal intensity drift over time. In addition, the relative standard deviations of the metabolic features were calculated across all QC samples, and those >30% were then removed. Student's *t* tests were conducted to detect differences in metabolite concentrations between two groups. Partial least squares discriminant analysis (PLS‐DA) was performed (R software, metaX package, function plot PLSDA) after log transformation and UV scaling (Wen et al., 2017). The altered metabolites between the two groups were identified by variable importance in the projection (VIP) >1 from PLS‐DA and statistical analysis (*p* < 0.05). Moreover, 200 permutation tests were used to investigate the quality of the model.

The altered metabolites were analysed by metabolomics pathway analysis (http://www.metaboanalyst.ca/) and were related to potential pathways (Pang et al., 2020). The KEGG database (http://www.kegg.jp/) was used to identify the function of these metabolites in various metabolic pathways.

### 4.7 Transcriptomic data sources and joint analysis of metabolites and genes

The rat RNA‐seq transcriptomics data, which included 4 young (21 weeks) male rats and 4 old (104 week) male rats, were downloaded from the Gene Expression Omnibus (GEO, http://www.ncbi.nlm.nih.gov/geo/) database (accession number GSE53960). Edge R was used for differential gene expression. The criteria of differentially expressed genes (DEGs) were |log_2_fold change| >1 and False Discovery Rate (FDR) <0.05. Metscape, a Cytoscape plugin (Gao et al., 2010; Shannon et al., 2003), was used to analyse the connected networks of metabolites and genes.

### 4.8 Statistical analysis

Two‐tailed Student's *t* test was used to analyse the significance between two groups, and one‐way ANOVA was used for comparison among more than two groups. A *p* value <0.05 indicated significance, and NS represented no significance.

## CONFLICT OF INTEREST

The authors declared that no competing interests exist.

## AUTHOR CONTRIBUTIONS

LJZ, JMS and YMY designed the project. QHH, HL, MYJ, YLZ and MLZ performed the experiment. QHH and LJZ performed data analysis with assistance from LW and ZXM. LJZ, JMS and QHH wrote the manuscript. YMY, QZ and ZXM contributed to discussion, review and edit the manuscript.

## Supporting information

Fig S1

Fig S2

Fig S3

Fig S4

Fig S5

Fig S6

Fig S7

Fig S8

Tab S1‐S2

Tab S3

Movie S1

Movie S2

## Data Availability

All untargeted metabolomic data used in this publication have been deposited to the EMBL‐EBI MetaboLights database with the identifier MTBLS2361 (liver metabolomics) and MTBLS2717 (serum metabolomics). The complete data set can be accessed at https://www.ebi.ac.uk/metabolights/MTBLS2361 and https://www.ebi.ac.uk/metabolights/MTBLS2717.
