# Supplementary figures and images for: Age‐related changes in metabolites in young donor livers and old recipient sera after liver transplantation from young to old rats
Source: Aging Cell. 2021 Jun 22;20(7):e13425. doi: 10.1111/acel.13425 (PMC8282239; doi:10.1111/acel.13425)

(a)

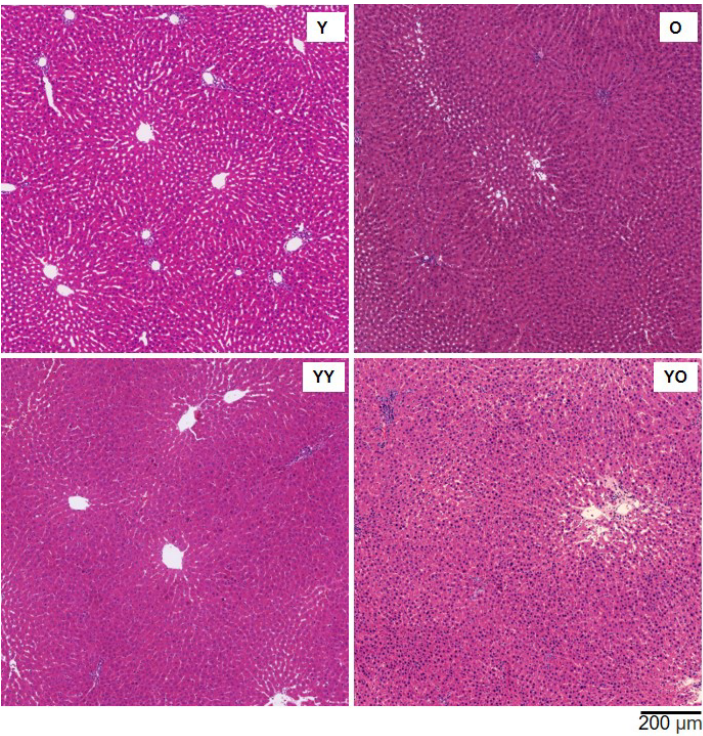

(b)

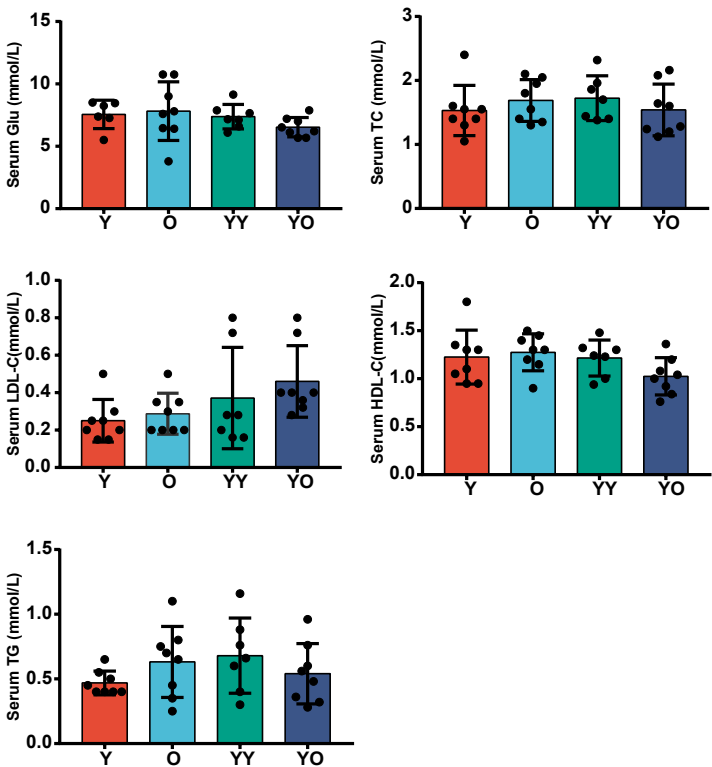

Supplement: Supplementary file 1 — Fig S1 [file ACEL-20-e13425-s009.pdf]

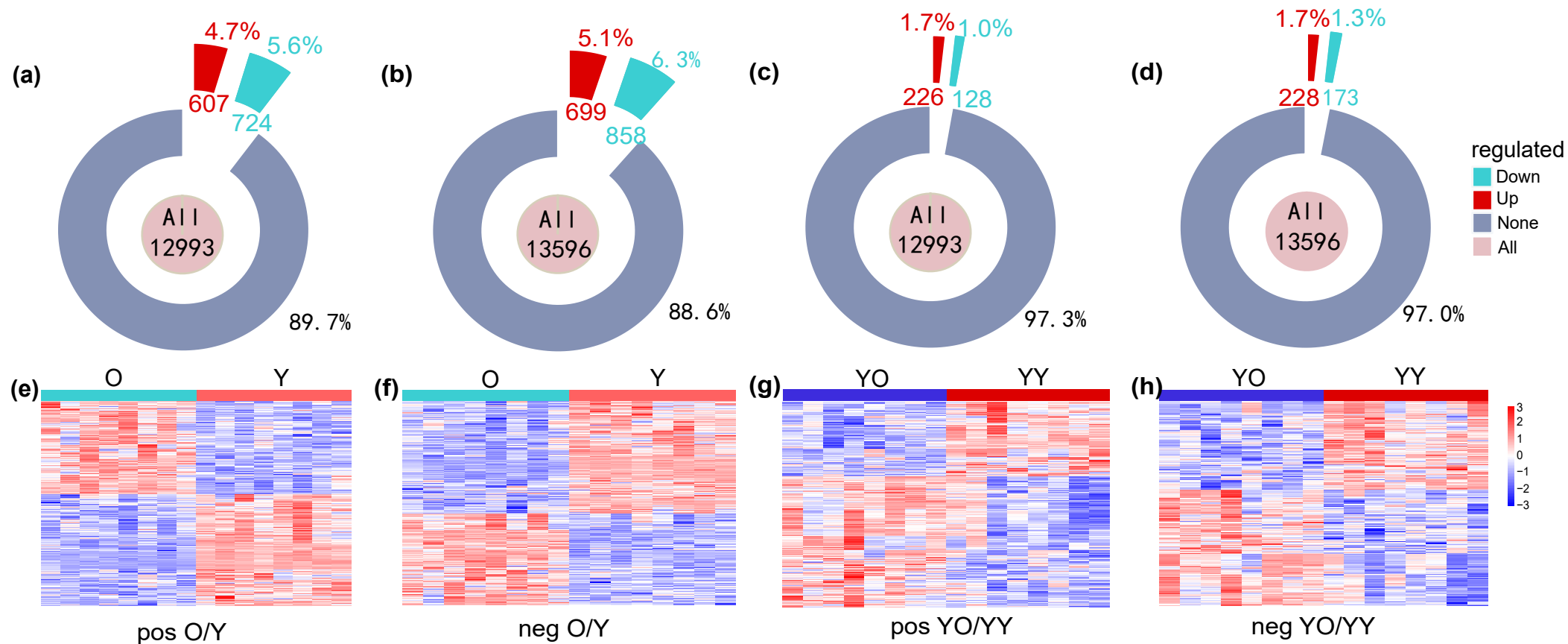

Supplement: Supplementary file 2 — Fig S2 [file ACEL-20-e13425-s011.pdf]

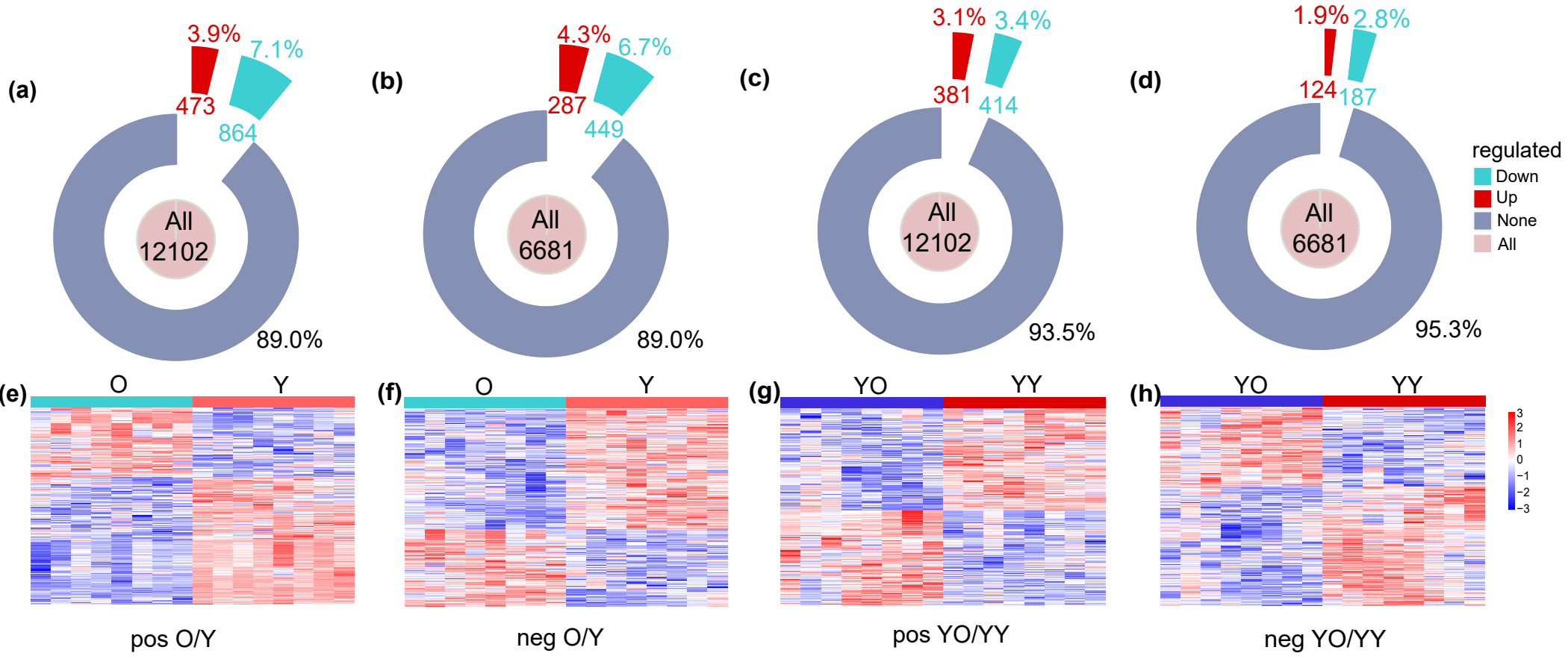

Supplement: Supplementary file 3 — Fig S3 [file ACEL-20-e13425-s006.pdf]

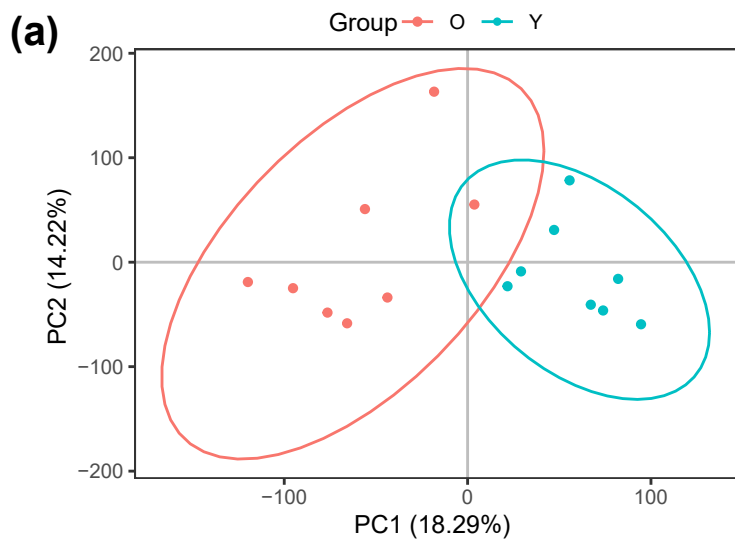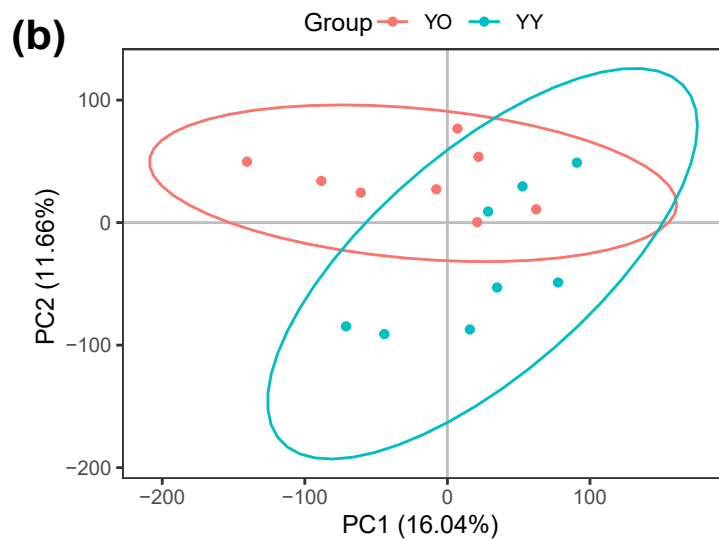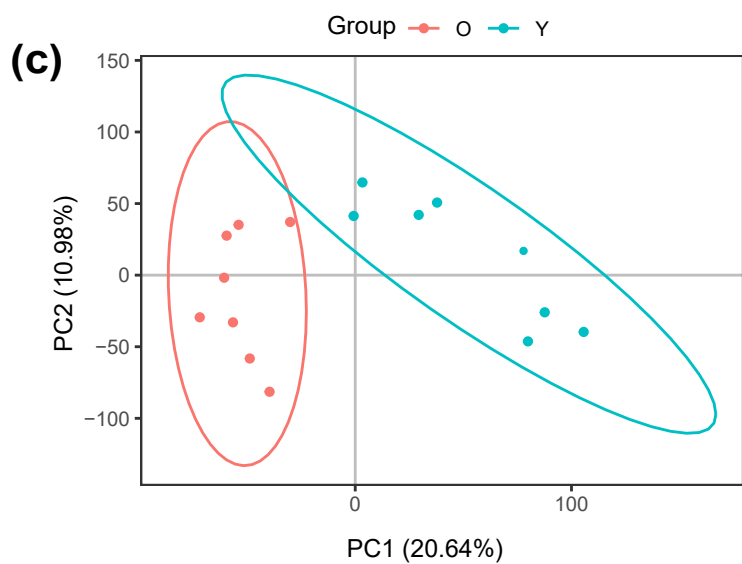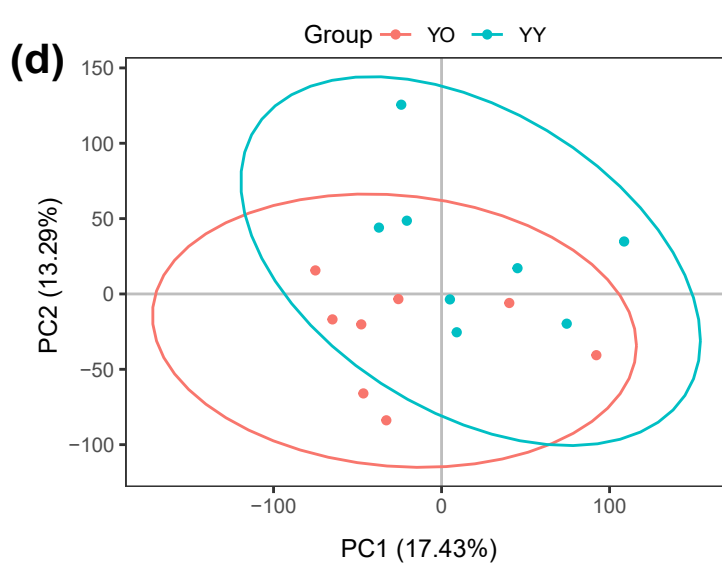

Supplement: Supplementary file 4 — Fig S4 [file ACEL-20-e13425-s003.pdf]

(a)

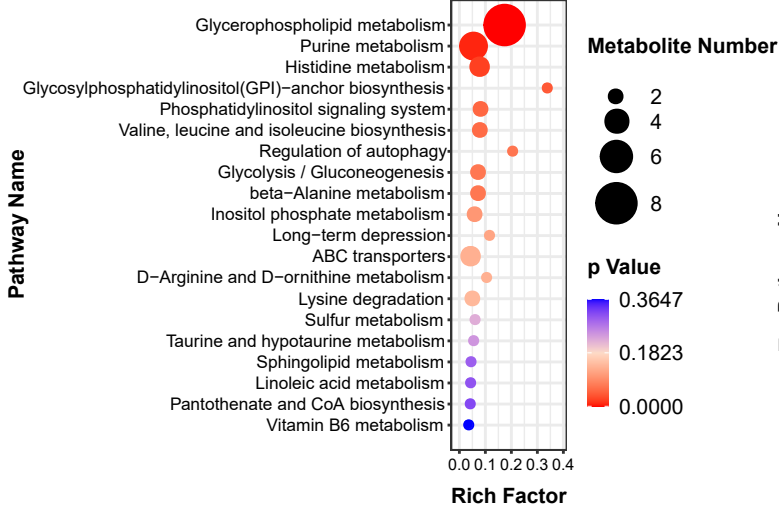

(b)

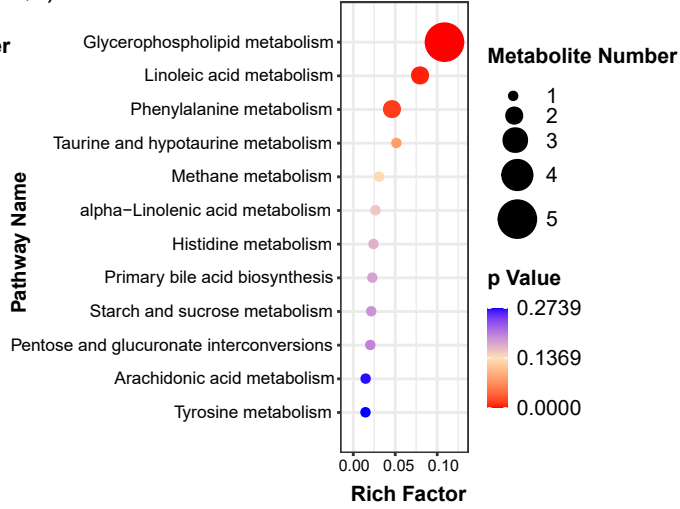

Supplement: Supplementary file 5 — Fig S5 [file ACEL-20-e13425-s012.pdf]

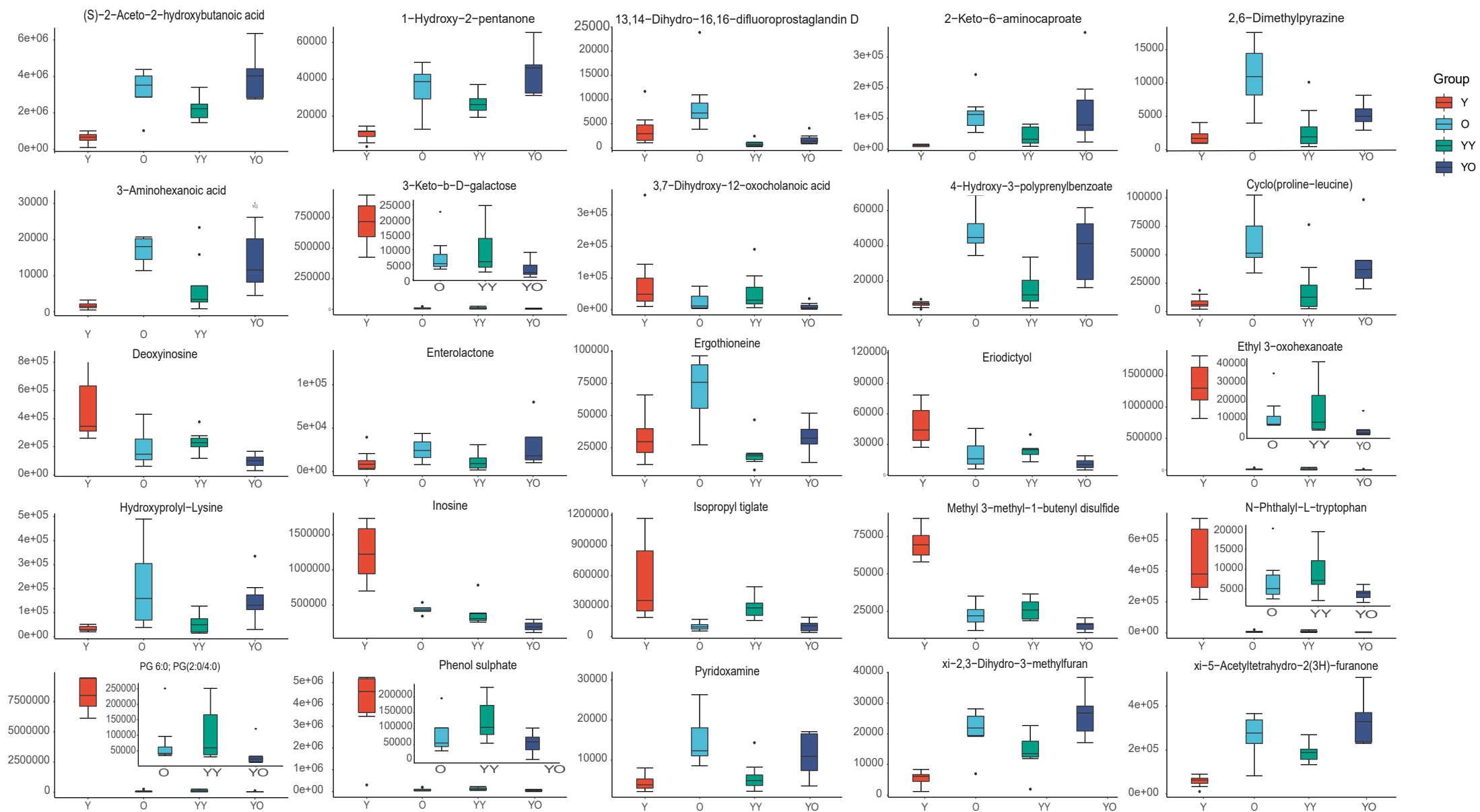

Supplement: Supplementary file 6 — Fig S6 [file ACEL-20-e13425-s004.pdf]

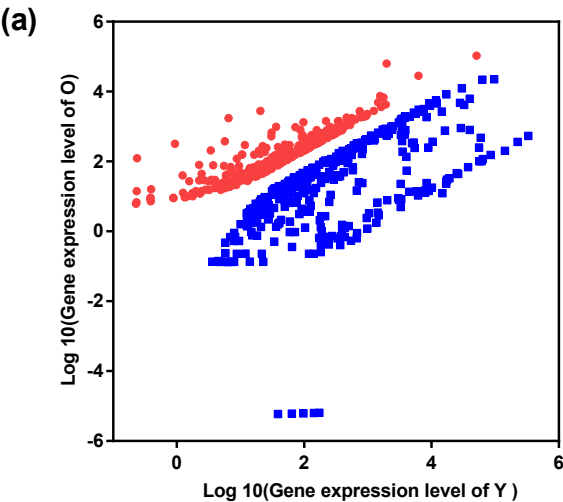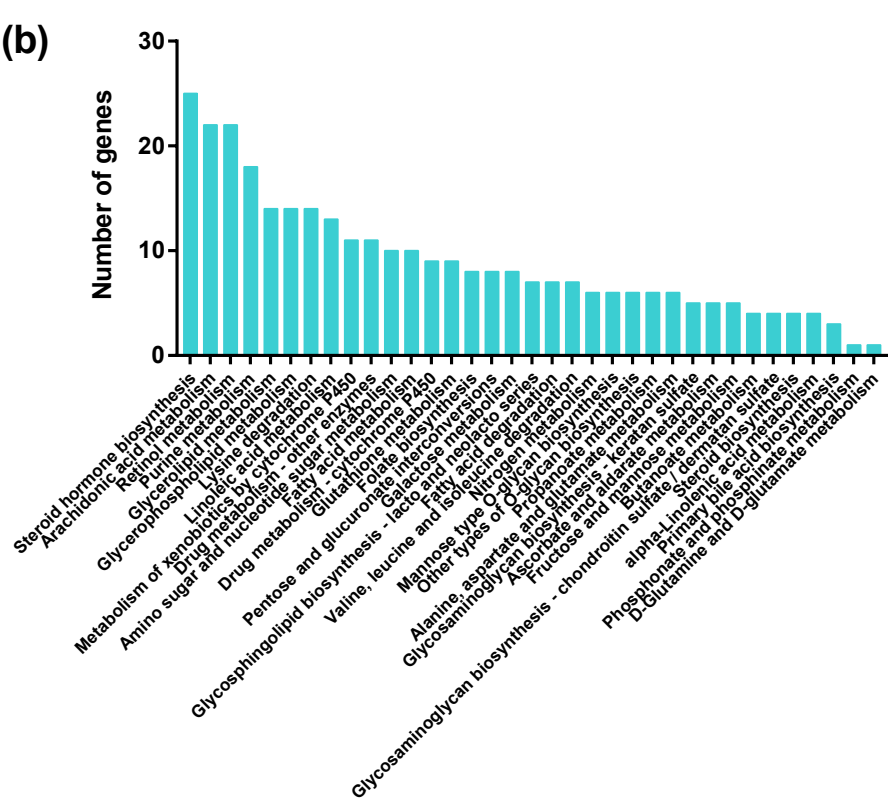

Supplement: Supplementary file 7 — Fig S7 [file ACEL-20-e13425-s010.pdf]

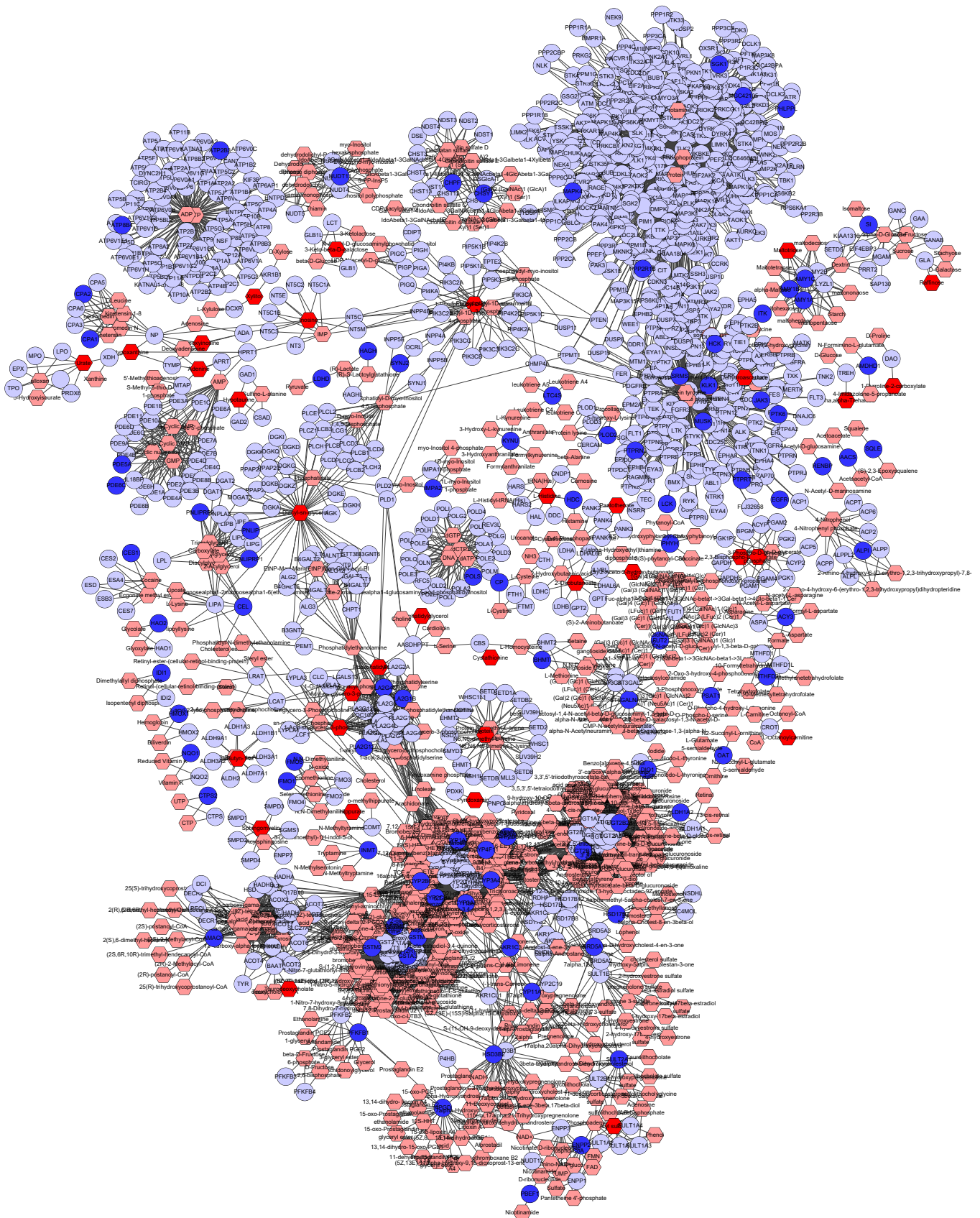

Supplement: Supplementary file 8 — Fig S8 [file ACEL-20-e13425-s002.pdf]
