# Supplementary material for: Age‐related changes in metabolites in young donor livers and old recipient sera after liver transplantation from young to old rats
Source: Aging Cell. 2021 Jun 22;20(7):e13425. doi: 10.1111/acel.13425 (PMC8282239; doi:10.1111/acel.13425)
Supplement: Supplementary file 9 — Tab S1‐S2 [file ACEL-20-e13425-s005.doc]

**Supplementary Information**

**Age-related changes in metabolites in young donor livers and old recipient sera after liver transplantation from young to old rats**

**Supplementary Experimental Procedures**

**1 |** **Histopathological analysis of liver tissue**

Dissected liver tissues were fixed in 4% paraformaldehyde solution and paraffin-embedded (FFPE). Sections (5μm thick) were stained with haematoxylin-eosin (HE), and images were captured by light microscopy (Eclipse E100, Nikon).

**2 | Serum biochemical analysis**

The serum TG (total triglyceride), TC (total cholesterol), LDL-C (low-density lipoprotein cholesterol), HDL-C (high-density lipoprotein cholesterol), and Glu (glucose) concentrations were measured by automatic biochemical analyzer (Mindray, BS220).

| **Table S1-1. Differential metabolites that were significant changed in O/Y of liver tissues** | | | | | |
| --- | --- | --- | --- | --- | --- |
| **Superclass** | **Class** | **Metabolite** | **Ratio** | **p.value** | **VIP** |
| Lipids and lipid-like molecules | Fatty Acyls | Isopropyl tiglate | 0.18 | 0.0004 | 2.73 |
|  |  | Octanoylcarnitine | 0.24 | 0.0006 | 2.62 |
|  |  | 2,2-Dimethylsuccinic acid | 0.40 | 0.0003 | 1.77 |
|  |  | 8-Isoprostaglandin E1 | 2.20 | 0.0007 | 1.29 |
|  |  | 17(18)-Epoxy-5Z,8Z,11Z,14Z-  eicosatetraenoic acid | 2.21 | 0.0031 | 1.35 |
|  |  | 2-Linoleoylglycerol | 2.39 | 0.0100 | 1.51 |
|  |  | Linoleoylcarnitine | 2.40 | 0.0116 | 1.18 |
|  |  | 8-iso-PGF3a | 2.42 | 0.0000 | 1.67 |
|  |  | Undecanedioic acid | 2.45 | 0.0040 | 1.61 |
|  |  | 13,14-Dihydro-15-keto PGF2a | 2.70 | 0.0005 | 1.70 |
|  |  | Acylcarnitine 15:0 | 2.79 | 0.0094 | 2.01 |
|  |  | Acylcarnitine 16:1 | 2.84 | 0.0018 | 2.13 |
|  |  | L-Propionylcarnitine | 3.14 | 0.0039 | 2.12 |
|  |  | Oleoyl-L-carnitine | 3.51 | 0.0005 | 2.19 |
|  |  | 10,11-dihydro-20-dihydroxy-LTB4 | 3.80 | 0.0000 | 1.85 |
|  | Glycerolipids | DG 40:8; DG(18:2/22:6) | 0.32 | 0.0497 | 1.49 |
|  | Glycerophospholipids | PG 6:0; PG(2:0/4:0) | 0.01 | 0.0000 | 4.70 |
|  |  | LysoPC 18:1 | 2.08 | 0.0029 | 1.35 |
|  |  | LysoPC 22:5 | 2.21 | 0.0384 | 1.47 |
|  |  | LysoPE 22:5 | 2.28 | 0.0075 | 1.48 |
|  |  | LysoPI 16:1; LysoPI 16:1 | 2.36 | 0.0081 | 1.35 |
|  |  | LysoPS 18:1; LysoPS 18:1 | 2.40 | 0.0204 | 1.10 |
|  |  | PC 20:3; PC(2:0/18:3) | 2.42 | 0.0459 | 1.57 |
|  |  | LysoPE 22:4 | 2.49 | 0.0055 | 1.87 |
|  |  | LysoPC 22:4 | 2.50 | 0.0010 | 1.76 |
|  |  | LysoPS 16:0; LysoPS 16:0 | 2.63 | 0.0178 | 1.59 |
|  |  | PI 36:4; PI(16:0/20:4) | 2.77 | 0.0393 | 1.42 |
|  |  | LysoPE 18:2 | 2.78 | 0.0016 | 1.96 |
|  |  | LysoPS 22:5; LysoPS 22:5 | 2.89 | 0.0040 | 1.44 |
|  |  | LysoPE 20:4 | 3.08 | 0.0005 | 2.30 |
|  |  | PC 19:1; PC(4:0/15:1) | 3.46 | 0.0208 | 1.71 |
|  |  | LysoPS 22:6; LysoPS 22:6 | 3.85 | 0.0012 | 1.85 |
|  |  | LysoPS 18:2; LysoPS 18:2 | 4.60 | 0.0004 | 1.93 |
|  |  | LysoPS 20:4; LysoPS 20:4 | 7.85 | 0.0008 | 2.64 |
|  | Prenol lipids | Geranylgeranylcysteine | 0.34 | 0.0112 | 1.64 |
|  |  | (S)-Oleuropeic acid | 4.04 | 0.0009 | 1.85 |
|  |  | 4-Hydroxy-3-polyprenylbenzoate | 7.10 | 0.0000 | 3.24 |
|  |  | Ginsenoside Rh1 | 0.50 | 0.0051 | 1.71 |
|  |  | beta-Santalyl acetate | 2.58 | 0.0117 | 2.33 |
|  | Sphingolipids | SM 34:1; SM(d18:1/16:0) | 4.91 | 0.0264 | 2.19 |
|  | Sterol Lipids | 7-Oxocholesterol | 0.28 | 0.0002 | 1.65 |
|  |  | 3,7-Dihydroxy-12-  oxocholanoic acid | 0.29 | 0.0467 | 1.78 |
|  |  | Taurodeoxycholic acid | 2.57 | 0.0270 | 1.43 |
|  |  | 19-Norepiandrosterone | 2.81 | 0.0002 | 2.29 |
|  |  | Beclomethasone | 0.44 | 0.0021 | 1.81 |
| Organic acids and derivatives | Carboxylic acids and derivatives | (S)-Homostachydrine | 0.12 | 0.0325 | 2.23 |
|  |  | DL-Cystathionine | 0.13 | 0.0000 | 3.19 |
|  |  | (+)-6-Aminopenicillanic acid | 0.20 | 0.0000 | 2.96 |
|  |  | Ne,Ne dimethyllysine | 0.24 | 0.0000 | 2.88 |
|  |  | D-1-[(3-Carboxypropyl)amino]-1-  deoxyfructose | 0.25 | 0.0003 | 2.80 |
|  |  | Stachydrine | 0.27 | 0.0018 | 2.38 |
|  |  | 2-Aminoisobutyric acid | 0.30 | 0.0000 | 2.74 |
|  |  | L-Histidine | 0.35 | 0.0003 | 1.90 |
|  |  | Ethenyl acetate | 0.36 | 0.0000 | 2.68 |
|  |  | Glycyl-Gamma-glutamate | 0.36 | 0.0015 | 2.07 |
|  |  | Glycyl-L-norleucine | 0.43 | 0.0003 | 1.70 |
|  |  | Propyl acetate | 0.46 | 0.0236 | 1.39 |
|  |  | Pantothenic acid | 0.47 | 0.0325 | 1.13 |
|  |  | Cystathionine ketimine | 0.48 | 0.0000 | 1.77 |
|  |  | N-lactoyl-Tryptophan | 0.49 | 0.0048 | 1.40 |
|  |  | Fructoseglycine | 0.50 | 0.0323 | 1.21 |
|  |  | Ergothioneine | 2.12 | 0.0060 | 1.67 |
|  |  | 1-Methyl-L-histidine | 2.12 | 0.0002 | 1.64 |
|  |  | Cyclo-prolylglycine | 2.60 | 0.0003 | 2.12 |
|  |  | (2-Methoxyethoxy)propanoic acid | 2.63 | 0.0077 | 1.36 |
|  |  | S-Carboxymethyl-L-cysteine | 3.00 | 0.0117 | 1.61 |
|  |  | Hydroxyprolyl-Lysine | 6.22 | 0.0029 | 2.39 |
|  |  | Cyclo(proline-leucine) | 7.71 | 0.0000 | 3.40 |
|  | Keto acids and derivatives | Ethyl 3-oxohexanoate | 0.01 | 0.0000 | 4.84 |
|  |  | 2-Ketobutyric acid | 0.14 | 0.0000 | 2.97 |
|  |  | 2-Oxosuccinamate | 0.37 | 0.0013 | 1.63 |
|  |  | Succinylacetone | 0.40 | 0.0015 | 1.91 |
|  |  | (S)-2-Aceto-2-hydroxybutanoic acid | 5.23 | 0.0001 | 2.90 |
|  |  | 2-Keto-6-aminocaproate | 9.71 | 0.0000 | 3.53 |
|  | Organic phosphoric acids and derivatives | Glyceric acid 1,3-biphosphate | 2.31 | 0.0072 | 1.32 |
|  | Organic sulfuric acids and derivatives | Phenol sulphate | 0.02 | 0.0000 | 4.00 |
|  |  | 3-[3-(Sulfooxy)phenyl]propanoic acid | 0.41 | 0.0243 | 1.73 |
|  | Sulfinic acids and derivatives | Hypotaurine | 0.48 | 0.0010 | 1.54 |
|  | Vinylogous thioesters | 4-Hydroxy-5-methyl-3(2H)-  thiophenone | 5.13 | 0.0000 | 2.71 |
| Organoheterocyclic compounds | - | 2-Aminobenzimidazole | 0.43 | 0.0286 | 1.53 |
|  | Azacyclic compounds | Dihydro-4,6-dimethyl-2-  (2-methylpropyl)-  4H-1,3,5-dithiazine | 0.31 | 0.0003 | 2.32 |
|  | Azoles | Allantoin | 24.65 | 0.0000 | 4.66 |
|  | Azolines | Parabanic Acid | 13.52 | 0.0001 | 3.46 |
|  | Diazines | 2,6-Dimethylpyrazine | 5.62 | 0.0000 | 2.79 |
|  | Dihydrofurans | 2-Nonenoic acid gamma-lactone | 2.94 | 0.0151 | 1.54 |
|  |  | xi-2,3-Dihydro-3-methylfuran | 3.91 | 0.0002 | 2.46 |
|  | Imidazopyrimidines | Adenine | 0.14 | 0.0347 | 2.20 |
|  |  | Hypoxanthine | 0.43 | 0.0000 | 2.11 |
|  |  | Uric acid | 5.15 | 0.0000 | 2.77 |
|  |  | Purine | 30.05 | 0.0000 | 4.31 |
|  | Indoles and derivatives | 1-Methyl-3-(2-thiazolyl)-1H-indole | 0.46 | 0.0032 | 1.53 |
|  |  | 1H-Indole-5-sulfonamide, N-(3-chlorophenyl)-3-  [[3,5-dimethyl-4-  [(4-methyl-1-piperazinyl)carbonyl]-  1H-pyrrol-2-yl]methylene]-2,3-  dihydro-N-methyl-2-oxo-, (3Z)- | 2.72 | 0.0058 | 1.71 |
|  | Lactones | Gamma-Butyrolactone | 0.34 | 0.0000 | 2.75 |
|  |  | Dehydroascorbic acid | 0.40 | 0.0122 | 1.71 |
|  |  | (xi)-(Z)-5-(3-Hexenyl)dihydro-  2(3H)-furanone | 3.14 | 0.0158 | 1.55 |
|  | Piperidines | Methyprylon | 2.17 | 0.0445 | 1.58 |
|  | Pyrazolopyrimidines | Allopurinol | 0.34 | 0.0000 | 2.24 |
|  | Pyridines and derivatives | 3-Pyridylacetic acid | 0.44 | 0.0235 | 1.35 |
|  |  | 3-Hydroxy-4-aminopyridine | 0.44 | 0.0106 | 1.57 |
|  |  | Pyridoxamine | 3.41 | 0.0000 | 2.44 |
|  |  | 2-Aminonicotinic acid | 4.62 | 0.0430 | 1.73 |
|  | Pyrroles | 1-Methylpyrrole | 0.44 | 0.0000 | 2.08 |
|  | Pyrrolidines | 2-Pyrrolidinone | 0.36 | 0.0001 | 2.69 |
|  | Pyrrolines | 1-Pyrroline-2-carboxylic acid | 0.40 | 0.0051 | 1.85 |
|  | Tetrahydroisoquinolines | Norsalsolinol | 3.14 | 0.0300 | 1.70 |
| Organic oxygen compounds | - | D-(+)-Raffinose | 0.02 | 0.0000 | 3.69 |
|  | Organooxygen compounds | D-myo-Inositol-1,2-diphosphate | 2.02 | 0.0133 | 1.30 |
|  | Organooxygen compounds | 3-Keto-b-D-galactose | 0.01 | 0.0000 | 4.81 |
|  |  | Maltotriose | 0.03 | 0.0000 | 3.85 |
|  |  | alpha.beta.-Trehalose | 0.12 | 0.0141 | 2.04 |
|  |  | 3'-Galactosyllactose | 0.30 | 0.0010 | 1.48 |
|  |  | 3-Butyn-1-al | 0.37 | 0.0000 | 2.65 |
|  |  | 2,3-Diphosphoglyceric acid | 2.12 | 0.0218 | 1.54 |
|  |  | D-glycero-L-galacto-Octulose | 2.75 | 0.0026 | 1.39 |
|  |  | Xylitol | 2.76 | 0.0041 | 1.32 |
|  |  | 1-Hydroxy-2-pentanone | 3.52 | 0.0001 | 2.44 |
|  |  | xi-5-Acetyltetrahydro-2(3H)-  furanone | 4.64 | 0.0001 | 2.75 |
| Organooxygen compounds | Carbohydrates and carbohydrate  conjugates | 4-O-beta-D-Galactopyranosyl-  D-xylose | 0.03 | 0.0000 | 4.00 |
| Benzenoids | - | 5-Methyl-1H-benzotriazole | 3.73 | 0.0002 | 2.63 |
|  | Benzene and  substituted derivatives | Hippuric acid | 0.40 | 0.0025 | 1.58 |
|  |  | 4-Phenyl-2-butenal | 2.03 | 0.0162 | 1.13 |
|  |  | 3-Methyl-1-phenyl-3-pentanol | 2.25 | 0.0000 | 2.02 |
|  |  | 4-Methylbenzoic acid | 30.84 | 0.0000 | 4.64 |
|  | Naphthalenes | 2-(4-Methoxyphenyl)  naphthalic anhydride | 0.19 | 0.0001 | 2.55 |
| Phenylpropanoids and polyketides | Flavonoids | Apigenin-6-C-glucoside-  7-O-glucoside | 0.06 | 0.0000 | 3.16 |
|  |  | Eriodictyol | 0.37 | 0.0046 | 1.99 |
|  |  | 3'-Hydroxy-4',5',7,8-  tetramethoxyflavone | 0.37 | 0.0001 | 1.22 |
|  |  | (S)-5,7-Dihydroxy-6,8-  dimethylflavanone | 3.52 | 0.0024 | 1.90 |
| Nucleosides, nucleotides, and analogues | Purine nucleosides | Inosine | 0.35 | 0.0000 | 1.94 |
|  |  | Deoxyinosine | 0.41 | 0.0050 | 1.80 |
| Organosulfur compounds | Organic disulfides | Methyl 3-methyl-1-  butenyl disulfide | 0.32 | 0.0000 | 2.52 |
| Alkaloids and derivatives | Tropane alkaloids | Calystegin A3 | 0.43 | 0.0044 | 1.85 |
| Lignans, neolignans and related compounds | Furanoid lignans | Enterolactone | 2.10 | 0.0270 | 1.66 |
| - | - | 2'-Hydroxydihydrodaidzein | 0.37 | 0.0000 | 2.31 |
|  |  | 3-Hydroxynonanoic acid | 4.00 | 0.0013 | 2.06 |
|  |  | N-Phthalyl-L-tryptophan | 0.02 | 0.0000 | 4.71 |
|  |  | Phenyl glucuronide | 0.12 | 0.0028 | 2.36 |
|  |  | Dehydro-L-(+)-ascorbic acid dimer | 0.20 | 0.0008 | 2.38 |
|  |  | N-(3-(Aminomethyl)  benzyl)acetamidine | 0.22 | 0.0010 | 2.30 |
|  |  | N-Carboxyethyl-.gamma.-  aminobutyric acid | 0.34 | 0.0000 | 1.79 |
|  |  | N-n-Butylpropionamide | 0.39 | 0.0013 | 2.18 |
|  |  | N-Formylglycine | 0.42 | 0.0001 | 1.55 |
|  |  | 13,14-Dihydro-15-  ketoprostaglandin A2 | 2.15 | 0.0010 | 1.32 |
|  |  | 9-Oxoprosta-10,12Z,14E-  trienoic acid | 2.16 | 0.0079 | 1.40 |
|  |  | 19(R)-Hydroxyprostaglandin B2 | 2.16 | 0.0181 | 1.15 |
|  |  | 13,14-Dihydro-16,16-  difluoroprostaglandin D2 | 2.30 | 0.0169 | 1.44 |
|  |  | 9-Oxo-10(E),12(E)-octadecadienoic acid | 2.50 | 0.0004 | 2.16 |
|  |  | 1,2-Dimethylimidazole | 2.53 | 0.0000 | 2.09 |
|  |  | 2,3-Diethylpyrazine | 2.64 | 0.0002 | 2.39 |
|  |  | Pyrimidinol | 2.90 | 0.0038 | 2.47 |
|  |  | 15-Ketoprostaglandin A1 | 6.16 | 0.0001 | 2.69 |
|  |  | 3-Aminohexanoic acid | 10.82 | 0.0000 | 3.80 |
|  |  | Castanospermine | 9.52 | 0.0000 | 3.67 |

| **Table S1-2. Differential metabolites that were significant changed in YO/YY of liver tissues** | | | | | |
| --- | --- | --- | --- | --- | --- |
| **Superclass** | **Class** | **Metabolite** | **Ratio** | **p.value** | **VIP** |
| Lipids and lipid-like molecules | Fatty Acyls | Isopropyl tiglate | 0.35 | 0.0007 | 2.92 |
|  |  | Acylcarnitine 7:0 | 0.39 | 0.0165 | 2.32 |
|  | Glycerophospholipids | PG 6:0; PG(2:0/4:0) | 0.32 | 0.0158 | 1.55 |
|  |  | LysoPC 20:1 | 2.36 | 0.0461 | 1.99 |
|  |  | CPA(18:0/0:0) | 2.62 | 0.0175 | 3.07 |
|  | Prenol lipids | Annocherin A | 0.36 | 0.0483 | 1.92 |
|  |  | 4-Hydroxy-3-polyprenylbenzoate | 2.53 | 0.0068 | 2.37 |
|  | Sterol Lipids | 3,7-Dihydroxy-12-oxocholanoic acid | 0.19 | 0.0109 | 3.03 |
|  |  | Glycocholic acid | 0.22 | 0.0210 | 2.56 |
| Organic acids and derivatives | Carboximidic acids and derivatives | L-Glutathione (oxidized form) | 7.76 | 0.0411 | 2.56 |
|  | Carboxylic acids and derivatives | L-Cysteine Sulfinic acid | 0.41 | 0.0048 | 2.29 |
|  |  | Cyclo(proline-leucine) | 2.00 | 0.0270 | 2.56 |
|  |  | L-Norleucine | 2.26 | 0.0236 | 1.77 |
|  |  | Hydroxyprolyl-Lysine | 2.77 | 0.0119 | 2.53 |
|  |  | Glutathione, oxidized | 7.03 | 0.0410 | 2.56 |
|  | Keto acids and derivatives | Ethyl 3-oxohexanoate | 0.26 | 0.0098 | 1.83 |
|  |  | 2-Keto-6-aminocaproate | 3.04 | 0.0272 | 2.45 |
|  | Organic sulfuric acids and derivatives | Phenol sulphate | 0.43 | 0.0410 | 1.81 |
|  |  | Ethyl sulfate | 4.56 | 0.0030 | 3.40 |
| Nucleosides, nucleotides, and analogues | Purine nucleosides | Deoxyinosine | 0.43 | 0.0018 | 2.34 |
|  | Purine nucleotides | Inosine 5'-monophosphate | 0.29 | 0.0003 | 3.75 |
|  | Pyrimidine nucleotides | Thymidine 5'-monophosphate | 0.50 | 0.0001 | 2.41 |
| Organoheterocyclic compounds | Dioxolanes | 1,1'-(Tetrahydro-6a-hydroxy-  2,3a,5-trimethylfuro[2,3-d]-  1,3-dioxole-2,5-diyl) bis-ethanone | 0.41 | 0.0214 | 1.36 |
|  | Indoles and derivatives | Luzindole | 2.10 | 0.0367 | 1.27 |
|  |  | 1H-Indole-3-carboxaldehyde | 2.91 | 0.0401 | 2.05 |
|  | Pyridines and derivatives | Pyridoxamine | 2.19 | 0.0292 | 2.08 |
| Organic oxygen compounds | Organooxygen compounds | 3-Keto-b-D-galactose | 0.37 | 0.0303 | 1.46 |
| Phenylpropanoids and polyketides | Flavonoids | Eriodictyol | 0.46 | 0.0021 | 2.05 |
| Benzenoids | Benzene and substituted derivatives | Benzidine | 0.42 | 0.0419 | 1.88 |
| Lignans, neolignans and related compounds | Furanoid lignans | Enterolactone | 3.22 | 0.0369 | 2.62 |
| - | - | N-Phthalyl-L-tryptophan | 0.42 | 0.0247 | 1.21 |

| **Table S2-1. Differential serum metabolites that were significant changed in O/Y** | | | | | |
| --- | --- | --- | --- | --- | --- |
| **Superclass** | **Class** | **Metabolite** | **Ratio** | **p.value** | **VIP** |
| Lipids and lipid-like molecules | Fatty Acyls | Docosahexaenoic acid methyl ester | 0.18 | 0.0158 | 2.12 |
|  |  | 15,16-DiHODE | 0.22 | 0.0000 | 2.58 |
|  |  | 2-Nonenoic acid | 0.34 | 0.0009 | 1.58 |
|  |  | 9,10-Epoxyoctadecenoic acid | 0.34 | 0.0003 | 1.94 |
|  |  | Undecanoic acid | 0.39 | 0.0073 | 1.22 |
|  |  | 5-Hexyltetrahydro-2-furanoctanoic acid | 0.47 | 0.0041 | 1.83 |
|  |  | (9S,10S)-9,10-  dihydroxyoctadecanoate | 0.47 | 0.0114 | 1.50 |
|  |  | cis-5-Decenedioic acid | 0.48 | 0.0039 | 1.17 |
|  |  | Oleamide | 2.15 | 0.0026 | 1.60 |
|  | Glycerophospholipids | 1-(1Z-Octadecenyl)-sn-glycero-  3-phosphocholine | 0.24 | 0.0014 | 1.66 |
|  |  | LysoPC 18:0 | 0.28 | 0.0024 | 1.77 |
|  |  | LysoPC 17:1 | 0.40 | 0.0081 | 1.62 |
|  |  | LysoPE 18:3 | 0.44 | 0.0084 | 1.35 |
|  |  | LysoPI 16:0; LysoPI 16:0 | 2.60 | 0.0436 | 1.32 |
|  |  | PC(20:2(11Z,14Z)/20:  5(5Z,8Z,11Z,14Z,17Z)) | 3.92 | 0.0035 | 2.17 |
|  |  | LysoPS 20:4; LysoPS 20:4 | 5.53 | 0.0023 | 1.96 |
|  | Prenol lipids | 6,10,14-Trimethyl-5,9,13-  pentadecatrien-2-one | 0.28 | 0.0000 | 2.43 |
|  |  | (3beta,8beta)-3-Hydroxy-7(11)-  eremophilen-12,8-olide | 0.41 | 0.0288 | 1.41 |
|  |  | Sesquithujene | 3.65 | 0.0177 | 2.24 |
|  | Sterol Lipids | 7a,12a-Dihydroxy-3-oxo-4-cholenoic acid | 0.13 | 0.0023 | 2.40 |
|  |  | Beta-Cortol | 0.25 | 0.0021 | 1.59 |
|  |  | Ursocholic acid | 0.28 | 0.0121 | 2.11 |
|  |  | 3a,6b,7b,12a-Tetrahydroxy-  5b-cholanoic acid | 0.29 | 0.0085 | 2.16 |
|  |  | 7-Ketodeoxycholic acid | 0.32 | 0.0170 | 1.61 |
|  |  | 12-Ketodeoxycholic acid | 0.37 | 0.0181 | 1.13 |
|  |  | 3,7-Dihydroxy-12-oxocholanoic acid | 0.40 | 0.0198 | 1.48 |
|  |  | Deoxycholic acid | 0.42 | 0.0175 | 1.28 |
|  |  | 1b,3a,12a-Trihydroxy-5b-cholanoic acid | 0.43 | 0.0472 | 1.22 |
|  |  | Glycodeoxycholic acid | 5.63 | 0.0074 | 1.84 |
|  |  | N-[(3a,5b,7a)-3-hydroxy-24-oxo-7-  (sulfooxy)cholan-24-yl]-Glycine | 9.42 | 0.0059 | 1.82 |
|  |  | Tauro-b-muricholic acid | 12.73 | 0.0028 | 2.01 |
|  |  | Taurocholate | 13.56 | 0.0002 | 2.67 |
|  |  | Taurodeoxycholic acid | 16.25 | 0.0031 | 2.40 |
| Organoheterocyclic compounds | - | 5,7-dihydroxy-2-(4-hydroxyphenyl)-  3,6-dimethoxy-4H-chromen-4-one | 0.05 | 0.0000 | 3.74 |
|  | Azepines | 2,3,4,5,6,7-Hexahydro-6,7-  dimethylcyclopent[b]azepin-  8(1H)-one | 0.46 | 0.0087 | 1.17 |
|  | Benzopyrans | 3,4-Dihydro-2,2,5,7,8-  pentamethyl-2H-  1-benzopyran-6-ol | 0.27 | 0.0000 | 2.60 |
|  | Benzoxepines | Heliannuol C | 0.16 | 0.0001 | 3.11 |
|  | Heteroaromatic compounds | 2-Pentylfuran | 0.47 | 0.0205 | 1.25 |
|  | Indoles and derivatives | 3-Indoleacrylic acid | 0.13 | 0.0002 | 2.92 |
|  |  | 1H-Indole-3-propanoic acid | 0.19 | 0.0001 | 2.66 |
|  |  | Indoleacrylic acid | 0.32 | 0.0015 | 1.80 |
|  |  | cyclic 6-Hydroxymelatonin | 0.44 | 0.0051 | 1.33 |
|  | Oxanes | 2-Exo-hydroxy-1,8-cineole | 0.24 | 0.0013 | 1.86 |
|  |  | (1S,2S,4S,5R)-1,8-Epoxy-  p-menthane-2,5-diol | 0.39 | 0.0000 | 1.91 |
|  | Piperidines | Normeperidine | 0.24 | 0.0000 | 2.73 |
|  |  | 2,2,6,6-Tetramethyl-4-piperidinone | 16.23 | 0.0000 | 3.60 |
|  | Tetrahydrofurans | 2,5-Diethyltetrahydrofuran | 0.36 | 0.0002 | 1.73 |
| Organic acids and derivatives | Carboxylic acids and derivatives | 3-Methyl-L-histidine | 0.26 | 0.0093 | 1.96 |
|  |  | Aspartyl-Isoleucine | 0.34 | 0.0000 | 2.26 |
|  |  | 3-Methylbutyl 2-methylpropanoate | 0.43 | 0.0035 | 1.41 |
|  |  | Astin I | 3.60 | 0.0032 | 1.81 |
|  | Organic sulfuric acids and derivatives | 4-hydroxybenzoic acid-4-O-sulphate | 0.26 | 0.0013 | 2.11 |
|  |  | 2-aminophenol sulphate | 0.27 | 0.0418 | 1.42 |
|  |  | Phenol sulphate | 0.37 | 0.0114 | 1.77 |
|  |  | p-Cresol sulfate | 2.34 | 0.0372 | 1.19 |
| Organic oxygen compounds | Organooxygen compounds | 2,8-Dihydroxyquinoline-  beta-D-glucuronide | 0.05 | 0.0000 | 2.99 |
|  |  | Diphenol glucuronide | 0.06 | 0.0000 | 3.34 |
|  |  | 5-Hydroxy-6-methoxyindole  glucuronide | 0.06 | 0.0014 | 2.37 |
|  |  | 2-Acetyl-4-methylpyridine | 0.13 | 0.0000 | 2.94 |
|  |  | Hexaethylene glycol | 0.31 | 0.0000 | 2.40 |
| Phenylpropanoids and polyketides | Cinnamic acids and derivatives | 2-Hydroxycinnamic acid | 0.31 | 0.0021 | 2.02 |
|  | Coumarins and derivatives | Umbelliferone | 0.49 | 0.0100 | 1.59 |
|  | Flavonoids | Quercetin 3,7-dimethyl ether | 0.11 | 0.0000 | 3.29 |
|  |  | Hecogenin | 4.00 | 0.0033 | 2.24 |
|  | Stilbenes | Benzoin | 0.12 | 0.0003 | 3.55 |
| Benzenoids | Benzene and substituted derivatives | Hippuric acid | 0.11 | 0.0000 | 3.20 |
|  |  | 2,6-Dihydroxybenzoic acid | 0.36 | 0.0030 | 1.53 |
|  | Phenols | Phenol | 0.40 | 0.0135 | 1.70 |
|  |  | 2,2'-Methylene-bis(6-tert-butyl-4 methylphenol) | 10.55 | 0.0000 | 3.81 |
| Alkaloids and derivatives | Harmala alkaloids | 3-Carboxy-2,3,4,9-tetrahydro-1H  -pyrido[3,4-b]indole-1-propanoic acid | 0.28 | 0.0000 | 1.87 |
|  | Tropane alkaloids | Physoperuvine | 0.45 | 0.0009 | 1.16 |
| Organic nitrogen compounds | Organonitrogen compounds | Trimethylamine N-oxide | 0.39 | 0.0024 | 1.81 |
| - | - | 2,6-Dimethyl-6-hepten-1-ol | 0.45 | 0.0000 | 1.76 |
|  |  | Tetraethylene glycol | 0.25 | 0.0000 | 2.66 |
|  |  | Tetrahydrothiophene sulfoxide | 0.26 | 0.0001 | 2.36 |
|  |  | 9-(Benzylamino)-1,2,3,4  -tetrahydroacridin-1-ol | 0.28 | 0.0000 | 2.52 |
|  |  | Pentaethylene glycol | 0.28 | 0.0000 | 2.47 |
|  |  | Hexadecanedioic acid,  3,3,14,14-tetramethyl- | 0.36 | 0.0000 | 1.54 |
|  |  | Phenyl glucuronide | 0.40 | 0.0195 | 1.64 |
|  |  | 1-Aminocyclohexanecarboxylic acid | 0.42 | 0.0347 | 1.58 |
|  |  | Valproic acid .beta.-D-glucuronide | 0.43 | 0.0500 | 1.43 |
|  |  | 13,14-Dihydro-16,16-  difluoroprostaglandin D2 | 4.85 | 0.0418 | 2.02 |
|  |  | L-Valinamide | 46.78 | 0.0011 | 3.96 |

| **Table S2-2.Differential serum metabolites that were significant changed in YO/YY** | | | | | | |  |
| --- | --- | --- | --- | --- | --- | --- | --- |
| **Superclass** | **Class** | **Metabolite** | **Ratio** | **p.value** | | **VIP** |  |
| Lipids and lipid-like molecules | Fatty Acyls | Oleic acid | 0.26 | 0.0270 | | 1.57 |  |
|  |  | 2-Hydroxymyristic acid | 0.31 | 0.0387 | | 2.22 |  |
|  |  | (9S,10E,12S,13S)-9,12,13-  Trihydroxy-10-octadecenoic acid | 0.45 | 0.0488 | | 1.63 |  |
|  |  | Docosahexaenoic acid methyl ester | 0.46 | 0.0201 | | 1.56 |  |
|  |  | Acylcarnitine 16:5 | 2.76 | 0.0008 | | 2.16 |  |
|  |  | Lauroyldiethanolamide | 4.01 | 0.0003 | | 2.65 |  |
|  | Glycerophospholipids | LysoPE 18:0 | 0.06 | 0.0161 | | 3.30 |  |
|  |  | LysoPC 20:1 | 0.21 | 0.0009 | | 3.36 |  |
|  |  | LysoPC 16:0 | 0.27 | 0.0166 | | 2.03 |  |
|  |  | PC(16:0/16:1(9Z)) | 0.29 | 0.0006 | | 2.41 |  |
|  |  | 1-Heptadecanoyl-sn-glycero-  3-phosphocholine | 0.31 | 0.0081 | | 2.67 |  |
|  |  | LysoPC 17:0 | 0.32 | 0.0401 | | 1.57 |  |
|  |  | LysoPC 18:1 | 0.34 | 0.0113 | | 2.51 |  |
|  |  | LysoPC 20:2 | 0.40 | 0.0311 | | 3.14 |  |
|  |  | 1-(1Z-Octadecenyl)-sn-glycero-  3-phosphocholine | 0.41 | 0.0190 | | 1.82 |  |
|  |  | PI 36:4; PI(16:0/20:4) | 3.07 | 0.0025 | | 2.19 |  |
|  |  | PC(18:2(9Z,12Z)/14:0) | 4.23 | 0.0020 | | 2.15 |  |
|  | Prenol lipids | (3beta,8beta)-3-Hydroxy-7(11)-  eremophilen-12,8-olide | 0.30 | 0.0005 | | 2.22 |  |
|  |  | Valerenic acid | 0.47 | 0.0050 | | 1.69 |  |
|  | Sterol Lipids | 7.alpha.-Hydroxy-4-  cholesten-3-one | 0.22 | 0.0007 | | 2.87 |  |
| Organic acids and derivatives | Carboxylic acids and derivatives | Oxalic acid | 0.01 | 0.0450 | | 3.10 |  |
|  |  | N-Acetyl-DL-valine | 0.47 | 0.0092 | | 1.57 |  |
|  |  | L-Glutamine | 0.49 | 0.0487 | | 2.32 |  |
|  |  | N-Lauroylglycine | 5.56 | 0.0009 | | 3.01 |  |
|  |  | N-Methyl-L-alanine | 47.72 | 0.0106 | | 3.24 |  |
|  | Organic phosphoric acids and derivatives | Ethylphosphate | 0.48 | 0.0313 | | 1.42 |  |
| Organoheterocyclic compounds | Imidazopyrimidines | Adenine | 2.53 | 0.0152 | | 1.63 |  |
|  | Lactams | Epsilon-caprolactam | 0.42 | 0.0007 | | 1.98 |  |
|  | Oxocins | Heliannuol A | 0.32 | 0.0141 | | 2.11 |  |
|  | Pyridines and derivatives | Picolinic acid | 3.30 | 0.0031 | | 2.44 |  |
|  | Thiolanes | Dihydro-5-methyl-2(3H)-  thiophenone | 0.46 | 0.0472 | | 1.30 |  |
| Organic oxygen compounds | Carbonyl compounds | Isophorone | 2.16 | 0.0206 | | 1.94 |  |
|  | Organooxygen compounds | 2-trans-6-cis-Dodecadienal | 0.36 | 0.0075 | | 1.73 |  |
| Organic nitrogen compounds | Organonitrogen compounds | Trimethylamine N-oxide | 0.29 | | 0.0035 | 2.17 | |
| - | - | N-Ethylmaleimide | 0.03 | 0.0008 | | 4.09 |  |
|  |  | Cycloheptylamine | 0.24 | 0.0385 | | 2.05 |  |
|  |  | 2-Hexyl-4-pentynoic acid | 0.29 | 0.0429 | | 1.77 |  |
|  |  | 2'-Hydroxy-4'-  methoxyacetophenone | 0.45 | 0.0011 | | 2.17 |  |
|  |  | Kaurenic acid | 0.46 | 0.0442 | | 2.67 |  |
|  |  | DDAO | 2.06 | 0.0081 | | 1.68 |  |
|  |  | N-Methyl-.alpha.-aminoisobutyric acid | 2.18 | 0.0227 | | 1.42 |  |
|  |  | Erucamide | 3.11 | 0.0069 | | 2.21 |  |
|  |  | Palmitoyl sphingomyelin | 181.95 | 0.0089 | | 3.83 |  |

Supplemental figure legends

**Suppl. Fig. S1.** Histological staining of rat liver tissue (a) and serum parameters (b) before and after liver transplantation. (a) Representative pictures of HE staining of rat liver tissue. (b) Concentrations of Glu, TC, LDLC, HDLC and TG of rat sera. No significant differences were found between O and Y groups, YO and YY groups by two-tailed student’s t-test.

**Suppl. Fig. S2. Metabolic profiling of liver tissue for O/Y and YO/YY in positive and negative mode.** (a-d) Number and percentage of up-regulated (red), down-regulated (cyan) and no changed (grey) features. (e-h) Heatmap of significantly regulated features, color scale bar showed range of levels of metabolites, red indicated high, blue indicated low.

**Suppl. Fig. S3. Metabolic profiling of serum for O/Y and YO/YY in positive and negative mode.** (a-d) Number and percentage of up-regulated (red), down-regulated (cyan) and no changed (grey) features. (e-h) Heatmap of significantly regulated features, color scale bar showed range of levels of metabolites, red indicated high, blue indicated low.

**Suppl. Fig. S4. PCA plot based on all features of liver tissue and serum.** (a) Liver tissue between O and Y. (b) Liver tissue between YO and YY. (c) Serum between O and Y. (d) Serum between YO and YY. (a, c) showed a separated distribution between O and Y groups, (b, d) showed a separation but considerable overlapping between YO and YY groups.

**Suppl. Fig. S5. KEGG enrichment plot of differential metabolites of liver tissue and serum.** (a) KEGG plot of liver tissue between O and Y. (b) KEGG plot of serum between O and Y.

**Suppl. Fig. S6. Boxplot of 25 metabolites in liver tissues before and after OLT.** The LC-MS feature areas were used for boxplot.

**Suppl. Fig. S7. DEGs of liver tissues between O and Y.** (a) Scatter plot of DEGs, blue spots indicate down-DEGs, red spots indicate up-DEGs. (b) KEGG pathway enrichment in metabolism for DEGs. RNA-seq transcriptomics data were downloaded from GEO database (accession number GSE53960).

**Suppl. Fig. S8. Fully connected network of metabolites and genes.** The nodes in red indicated differential metabolites and the nodes in blue indicated differentially expressed genes. Hexagons represented metabolites, circles represented genes.
